# Endogenous “Time Bomb” – Mislocalized Phospholipase A2 as a Critical Mediator of Ultra‐Rapid Mortality in Sepsis and Acute Lung Injury

**DOI:** 10.1002/advs.202514915

**Published:** 2026-03-24

**Authors:** Jianyu Wang, Zhongxing Xu, Lin Wang, Xin Sui, Yuan Luo, Xiuli Zhao, Jun Yang, Yongan Wang

**Affiliations:** ^1^ Academy of Military Medical Sciences Beijing China; ^2^ Shenyang Pharmaceutical University Shenyang China

**Keywords:** acute lung injury, phospholipase A2, rapid mortality, sepsis, surface tension

## Abstract

This study reveals that phospholipase A2 (PLA2), normally stable and nontoxic, can be activated specifically within the alveolar environment to induce rapid, “electric shock‐like” lethality, akin to chemical toxins, while also exhibiting extreme toxicity comparable to that of biological toxins, and functioning as a potential “time bomb” in the body. When exacerbated inflammation impairs the pulmonary barrier, PLA2 from the circulation can penetrate into the lungs. Once activated in the alveolar space, it rapidly hydrolyzes pulmonary surfactant phospholipids, causing a drastic decline in surface tension (>30%). This leads to alveolar overdistension, instantaneous respiratory failure, and asphyxiation—an acute mortality effect strikingly similar to that observed in sepsis and severe pulmonary diseases. PLA2 penetration and lethality are more pronounced in aged animals. Based on these findings, a combination therapy comprising phospholipase (dioleoylphosphatidylserine) and an inhibitor (varespladib) was developed, which significantly improved survival rates from 0% to over 90% in mice with sepsis, acute lung injury, and PLA2 poisoning. This study provides critical theoretical foundations and intervention strategies for the clinical treatment of related diseases.

## Graphical abstract

Phospholipase A2 (PLA2), a dormant enzyme, becomes lethal when activated—collapsing lungs in minutes. Our dual therapy (DOPS + varespladib) boosts survival from 0% to >90% in sepsis/ALI. A breakthrough for acute lung injury treatment.

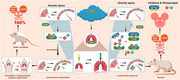

## 1 Introduction

Sepsis and severe pulmonary diseases are among the most critical conditions associated with acute mortality in humans [1, 2, 3]. Despite advancements in therapeutic strategies, including mechanical ventilation to maintain adequate oxygen supply and anti‐cytokine therapies to mitigate cytokine storms [4, 5, 6], mortality in the late stages of these diseases remains unpreventable. Conventional pathophysiological explanations often attribute this late‐stage mortality to systemic inflammatory response syndrome, metabolic collapse due to mitochondrial dysfunction, and coagulation disorders [7, 8, 9]. However, therapeutic strategies targeting these pathways have not significantly improved survival outcomes. Therefore, there is a pressing need to identify the underlying factors of sepsis and severe pulmonary disease that result in rapid and irreversible mortality.

Notably, the observation that acute intoxication [10, 11, 12] and fatal cases of severe pulmonary disease exhibited similar mortality rates and temporal patterns suggests a possible pathogenic association between them. This association prompted a hypothesis that an unrecognized terminal pathway, mediated by an endogenous substance that acquires toxin‐like lethality when activated in specific pathological conditions, may exist to lead the irreversible mortality of severe pulmonary disease. Whether natural or synthetic, the existence of substances that combine rapid lethality, including chemical toxins [13, 14, 15], with extreme toxicity, akin to biological toxins [16, 17], is a critical concern for public safety, protection measures, and early warning systems [18, 19, 20]. Most toxic substances are exogenous [21]. Moreover, under normal conditions, organisms do not endogenously produce, and secrete highly toxic compounds. However, after systematic screening of common inflammatory mediators, we report a substantial discovery: phospholipase A2 (PLA2) is stable and nontoxic under normal physiological conditions but can be activated under specific circumstances [22, 23, 24], triggering rapid, “electric shock‐like” death.

This finding indicates that PLA2 is the only known endogenous substance that exhibits both extreme toxicity and rapid lethality, and might explain the mechanisms underlying refractory respiratory failure and acute mortality at sepsis and severe pulmonary diseases. Based on this discovery, we developed and screened therapeutic drugs and administration strategies, offering a transformative approach to treating these severe diseases.

## 2 Results and Discussion

### 2.1 PLA2‐Induced Ultra‐Rapid Mortality

#### 2.1.1 Ultrapotent Toxicity of PLA2

**FIGURE 1:**
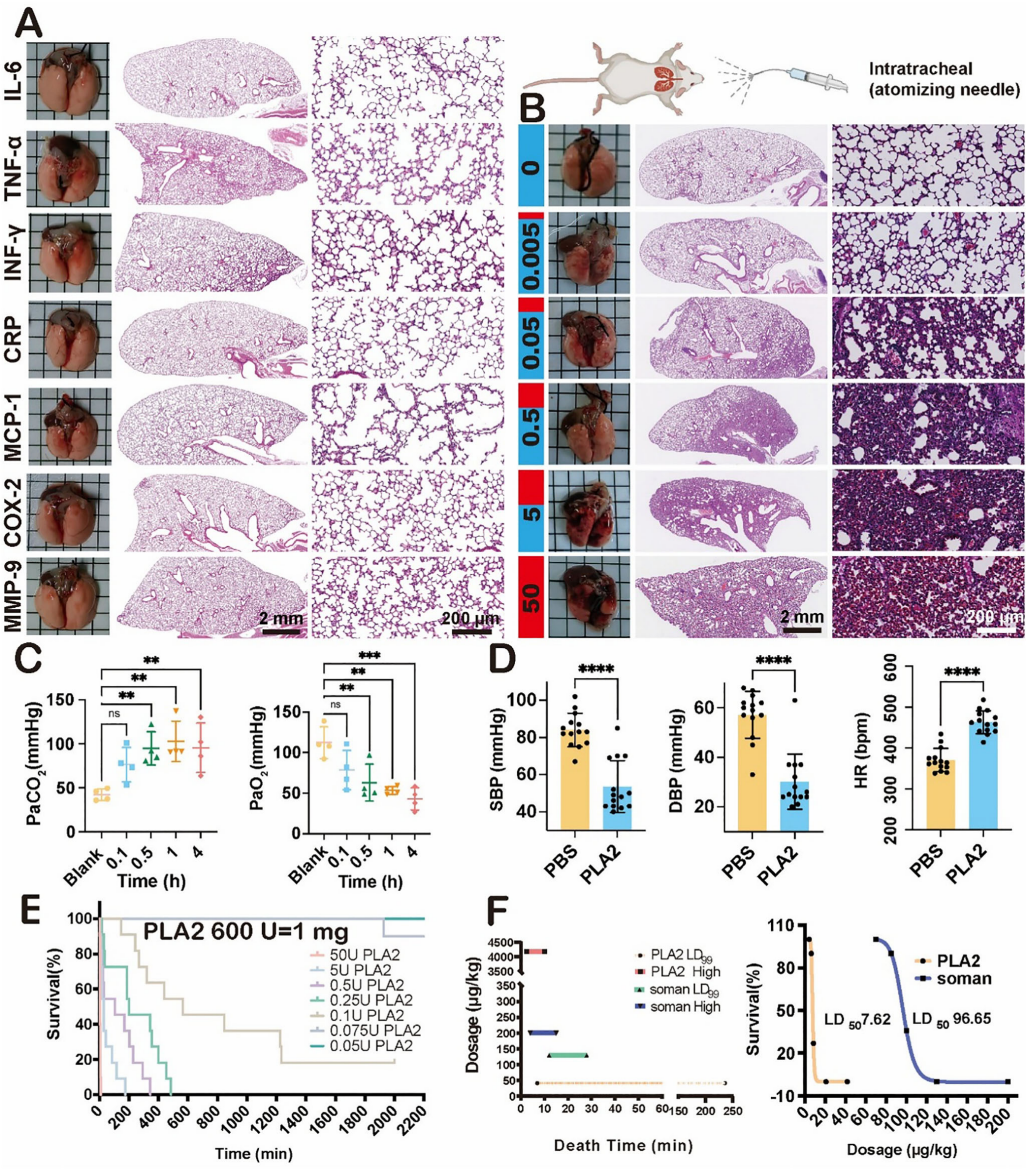
Mortality of PLA2. (A) Screening of different inflammatory mediators via intratracheal (IT) administration. (B) Lung photographs and H&E‐stained histopathology (mice treated with 0–50 U PLA2 via IT administration. (C) Arterial blood gas analysis (PLA2‐treated mice over 4 h): Left: PaO_2_; Right: PaCO_2_ (*n* = 4, ^**^ *p* < 0.01, ^***^ *p* < 0.001). (D) Hemodynamics (PLA2‐treated mice): Left: Systolic blood pressure (SBP); Middle: Diastolic blood pressure (DBP); Right: Heart rate (HR) (*n* = 14, ^****^ *p* < 0.0001). (E) Survival time (mice treated with PLA2) (*n* = 10). (F) PLA2 vs. soman lethality: Left: Time‐to‐death (low/high doses); Right: Dose‐dependent survival. PLA2, phospholipase A2; PBS, phosphate‐buffered saline.

Based on the clinical observation, it was hypothesized that an unrecognized terminal pathway mediated by an endogenous substance could acquire toxin‐like lethality when activated in specific pathologies. Systematic screening of common inflammatory mediators, including interleukin‐6 (IL‐6), tumor necrosis factor‐alpha (TNF‐α), interferon‐γ (IFN‐γ), C‐reactive protein (CRP), monocyte chemoattractant protein‐1 (MCP‐1), cyclooxygenase‐2 (COX‐2), matrix metalloproteinase‐9 (MMP‐9), and PLA2, via intratracheal (IT) administration into the lung revealed that most factors failed to cause mortality upon aerosolized exposure (Figure 1A). In stark contrast, only PLA2 (subtype III) induced severe pulmonary injury and death at ultralow doses. Intrapulmonary delivery of 0.05 U (83.5 ng) caused inflammation, alveolar collapse, interstitial edema (Figure 1B), hypoxemia (Figure 1C), increased heart rate, and decreased blood pressure (Figure 1D), while 0.5 U (835 ng) was 100% lethal in mice. A dose of 50 U (83.5 µg) caused death within 0.5–3 min due to complete respiratory arrest (Figure 1E; Video S1). Compared to soman—a fast‐acting nerve agent (Figure S1A) causing cholinergic crisis (Figure S2; Video S2) rather than direct lung injury (Figure S1B)—PLA2's median lethal dose (LD_50_ 7.62 µg/kg) was 13‐fold lower, and time to death was 10‐fold faster at high doses (Figure 1F) [25, 26]. PLA2 (LD_99_ 76 µg/kg) exhibits acute toxicity comparable to that of biotoxins, such as bungarotoxin (LD_99_ 90 µg/kg) and ricin (LD_99_ 10 µg/kg) [27, 28, 29], which are typically classified as subacute or chronic toxins due to their latency period of up to 24 h (Figure S3). Critically, unlike most toxins that require entry into the bloodstream to exert systemic effects, PLA2 uniquely caused rapid death via respiratory absorption alone.

#### 2.1.2 Species‐ and Isoform‐Specific Lethality of PLA2

Most known biotoxins derive from nonhuman species [30]. In contrast, this study uniquely identified endogenous humanized PLA2 accumulation during acute lung injury (ALI) and sepsis. Among humanized variants (Figure S4A), PLA2‐IIA caused 80% mortality within 40 min, while PLA2‐III/V and bee venom‐derived B‐III showed 100% mortality by 25–40 min (Figure S4B). Pathological severity was dependent on the PLA2 variant (Figure S4C), exhibiting dose‐dependent pulmonary damage (Figure S4D). Notably, PLA2‐IID caused pulmonary injury but was non‐lethal in animals. In addition to secretory PLA2s (GIII, GV, GIIA, and GIID), a systematic investigation was extended to other types, including Lp‐PLA2 (GXV/PAF‐AH, GVIIA), cPLA2 (GIVA), and iPLA2 (GVIA). Notably, none of these induced mortality or significant lung injury upon intratracheal instillation (Figure S5 and Table S1). Besides, to mitigate potential lung injury that could be confounded by impurities (such as endotoxin), all PLA2 used in this study were rigorously tested and confirmed to contain endotoxin levels well within the acceptable limits specified by pharmacopeial standards (Figure S6). Species‐dependent PLA2 toxicity was observed: humanized variants induced sialorrhea/oral foaming (absent with B‐III; Figure S4C), likely due to differential phospholipid (PL) targeting. Despite comparable toxicity between PLA2‐V and B‐III, the latter enzyme was selected for further study due to its commercial availability and cost efficiency.

#### 2.1.3 Route‐Dependent Distribution and Effect of PLA2

**FIGURE 2:**
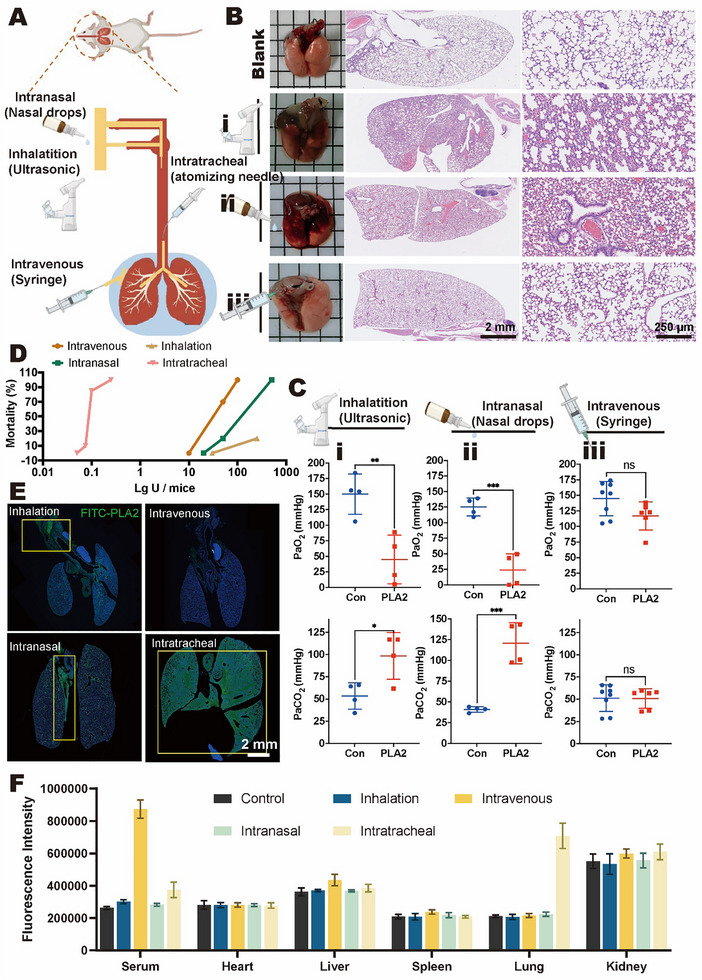
Different administration routes of PLA2. (A) Schematic of administration routes: intranasal (IN), inhalation (INH), IT, and intravenous (IV). (B) PLA2‐induced lung injury via INH (i), IN (ii), and IV (iii). Left: Photographs; Right: H&E histopathology. (C) Arterial blood gas analysis (PLA2‐treated mice): Top: PaCO_2_; Bottom: PaO_2_ (INH [i], IN [ii], IV [iii]) (*n* = 4, ^*^ *p* < 0.05, ^**^ *p* < 0.01, ^***^ *p* < 0.001). (D,E) Mortality (D) and fluorescein isothiocyanate (FITC)‐labeled PLA2 fluorescence. (E) across administration routes. Con, control. (F) Distribution of PLA2 in different organs via multiple administration routes (*n* = 8).

The acute lethality of PLA2 at ultralow doses was critically dependent on administration route (Figure 2A). Specifically, IT administration via atomizing needle—unlike inhalation (INH) or intranasal (IN) delivery—directly delivered PLA2 to the alveoli, causing rapid lethality (Figures 1 and 2D; Figure S3). Consistent with this route‐specific effect, INH delivery of a high concentration (1000 U/mL) caused only mild pulmonary damage (Figure 2Bi,Ci), whereas IN required a 5‐fold higher concentration to achieve lethality (Figure 2Bii,Cii,D). Intravenous (IV) administration showed no pulmonary effects (Figure 2Biii), indicating that systemic circulation does not mediate the acute lung injury. Fluorescence tracking confirmed route‐dependent lung distribution (Figure 2E), demonstrating that IT delivery effectively bypasses upper respiratory defenses. Furthermore, distinct PLA2 organ distribution patterns were observed: IT and INH administration drove accumulation in the lungs, while IV delivery led to enrichment in the liver (Figure 2F). Integrated analysis (Figures 1 and 2; Figure S3) revealed PLA2's context‐dependent toxicity: while systemically tolerated, its mislocalization to the alveolar space unlocks a latent lethal program, implicating endogenous PLA2 mislocalization as a plausible mechanism in cases of unexplained acute respiratory fatalities.

### 2.2 Pathogenic Mechanisms of PLA2‐Induced Ultra‐Rapid Mortality

#### 2.2.1 Conditions for PLA2‐Induced Apoptosis

**FIGURE 3:**
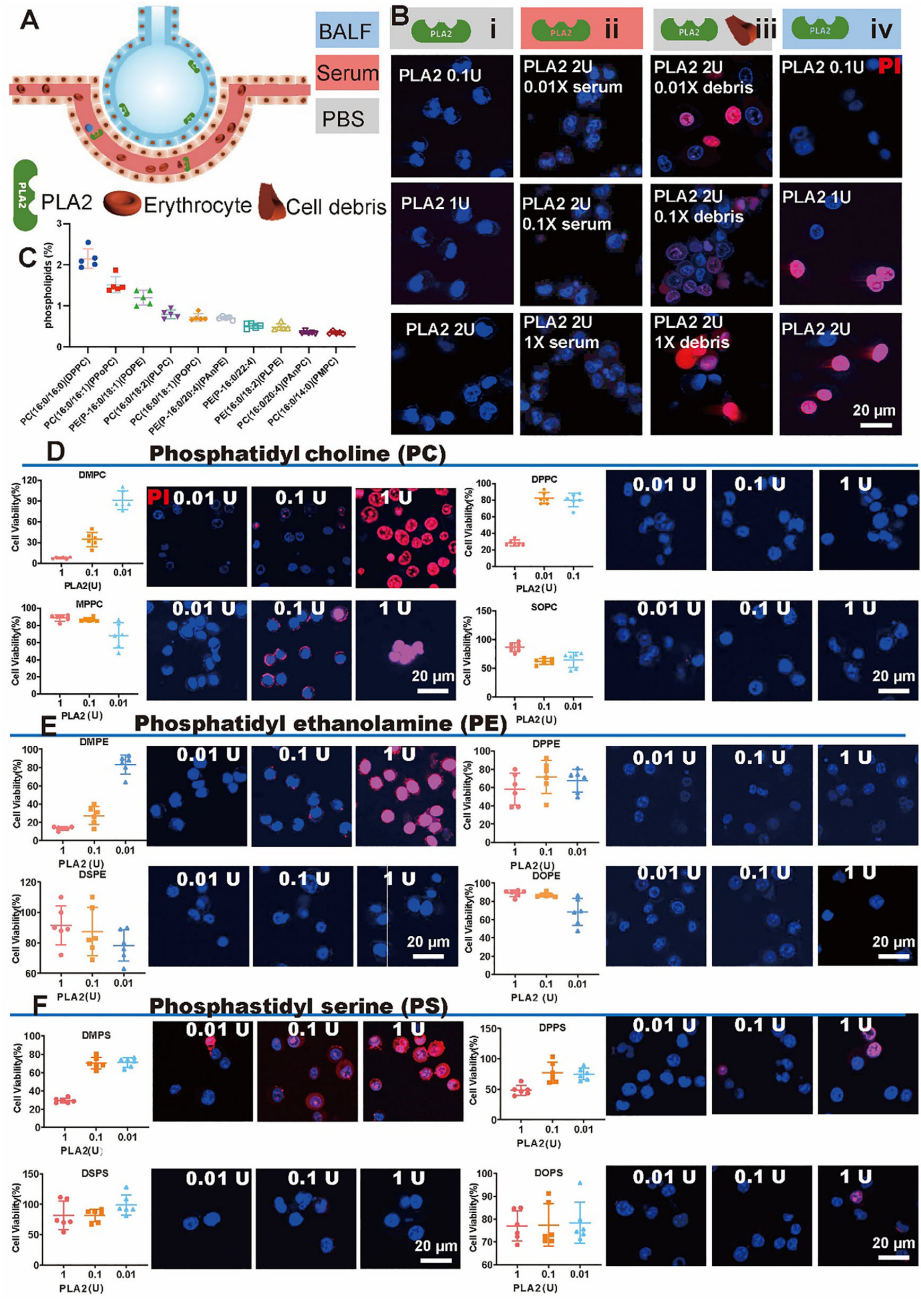
PL‐dependent PLA2 cytotoxicity mechanisms. (A) Schematic of microenvironments facilitating PLA2 activation. (B) PLA2 cytotoxicity under physiological variations assessed by PI fluorescence. (C) BALF PL composition (top 10) (*n* = 5). (D–F) PLA2 cytotoxicity via PC (D), PE (E), and PS (F) interactions: Left: Concentration‐dependent viability; Right: PI‐stained membrane damage. BALF, bronchoalveolar lavage fluid; DMPC, dimyristoylphosphatidylcholine; DPPC, dipalmitoylphosphatidylcholine; MPPC, 1‐myristoyl‐2‐palmitoylphosphatidylcholine; SOPC, 1‐stearoyl‐2‐oleoylphosphatidylcholine; DMPE, dimyristoylphosphatidylethanolamine; DPPE, dipalmitoylphosphatidylethanolamine; DSPE, distearoylphosphatidylethanolamine; DOPE, dioleoylphosphatidylethanolamine; DMPS, dimyristoylphosphatidylserine; DPPS, dipalmitoylphosphatidylserine; DSPS, distearoylphosphatidylserine; DOPS, dioleoylphosphatidylserine (*n* = 6).

The lung‐specific toxicity of PLA2 was investigated by comparing intra‐ and extra‐alveolar environments (Figure 3A). While PLA2 showed no cytotoxicity in PBS or serum (Figure 3Bi,ii), it induced significant membrane damage, as measured by propidium Iodide(PI) staining, in PL‐rich environments like cell debris or Bronchoalveolar lavage fluid (BALF) (Figure 3Biii,iv), demonstrating its microenvironment‐dependent toxicity [31, 32, 33]. To define the key component of this microenvironment, lipidomic analysis was performed, and identified phosphatidylcholine (PC) as the dominant pulmonary PL (Figure 3C). Subsequent studies on PLA2‐PL interactions revealed alkyl chain‐dependent non‐toxic (Figure 3D–F): myristoyl‐PLs (DMPC, DMPE, and DMPS) consistently caused damage regardless of head group, while oleoyl‐PLs remained safe (SOPC, DOPE, and DOPS). Notably, the highly abundant lung surfactant lipid DPPC showed minimal susceptibility to PLA2‐induced toxicity.

#### 2.2.2 PLA2‐PL Interaction‐Induced Lung Injury

**FIGURE 4:**
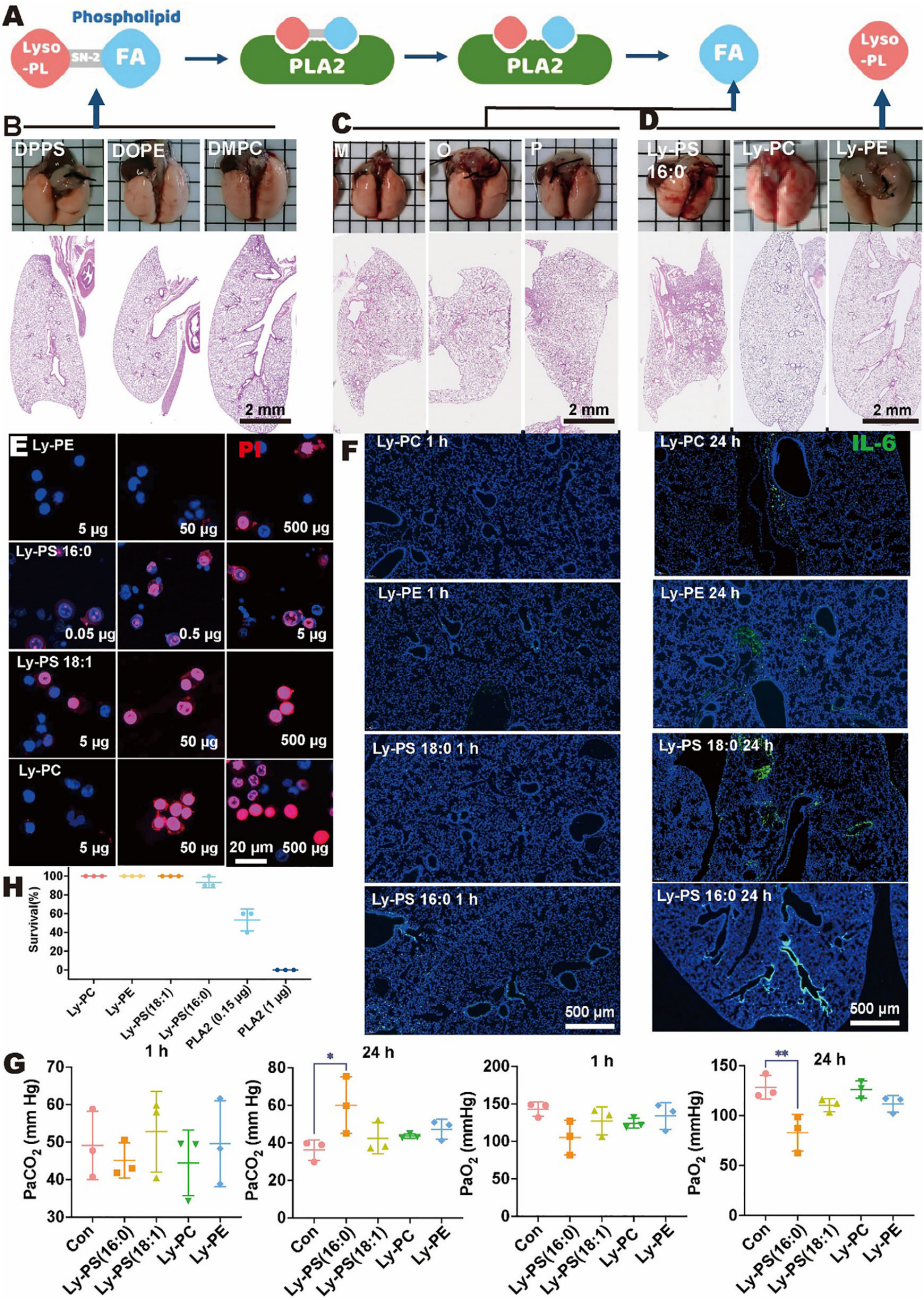
PLA2‐PL induced lung injury. (A) Schematic of PLA2‐mediated PL degradation. (B–D) Lung morphology after IT administration of PLs (B), FAs (Myristic acid, M; Oleic acid, O; Palmitic acid, P) (C), and lyso‐PLs (D). Top: Gross images; Bottom: Histopathology. (E) PI‐stained cellular fluorescence following lyso‐PL treatment. (F) IL‐6 immunofluorescence in lyso‐PL‐treated lungs at 1/24 h. (G) Arterial blood gas analysis post‐lyso‐PL administration: Left: PaCO_2_; Right: PaO_2_ (1/24 h) (*n* = 3, ^*^ *p* < 0.05, ^**^ *p* < 0.01). (H) Survival rates after PLA2/lyso‐PL IT administration. FA, fatty acid; lyso‐PL, lysophospholipid (*n* = 3).

PLA2 hydrolyzed PLs into lyso‐PLs and fatty acids (FAs, Figure 4A). A systematic analysis of these hydrolysis products revealed that the parent PLs and FAs showed minimal pathological effects (Figure 4B,C; Figure S7), In contrast, lyso‐PLs and particularly lyso‐PS (16:0), induced lung injury, characterized by alveolar collapse (Figure 4D), and epithelial cell apoptosis (Figure 4E). Furthermore, lyso‐PS administration triggered chronic inflammation, as evidenced by IL‐6 elevation and late‐onset hypoxia after 24 h (Figure 4F,G). Critically, however, even at a high dose (1 mg/mL), lyso‐PS failed to replicate the acute lethality observed with PLA2 itself (Figure 4H). These results collectively demonstrate that while PLA2‐generated lyso‐PLs contribute to lung injury, none of its hydrolysis products are sufficient to drive the rapid mortality directly caused by PLA2.

#### 2.2.3 PLA2‐Induced Hemolysis and Surface Tension Alterations

**FIGURE 5:**
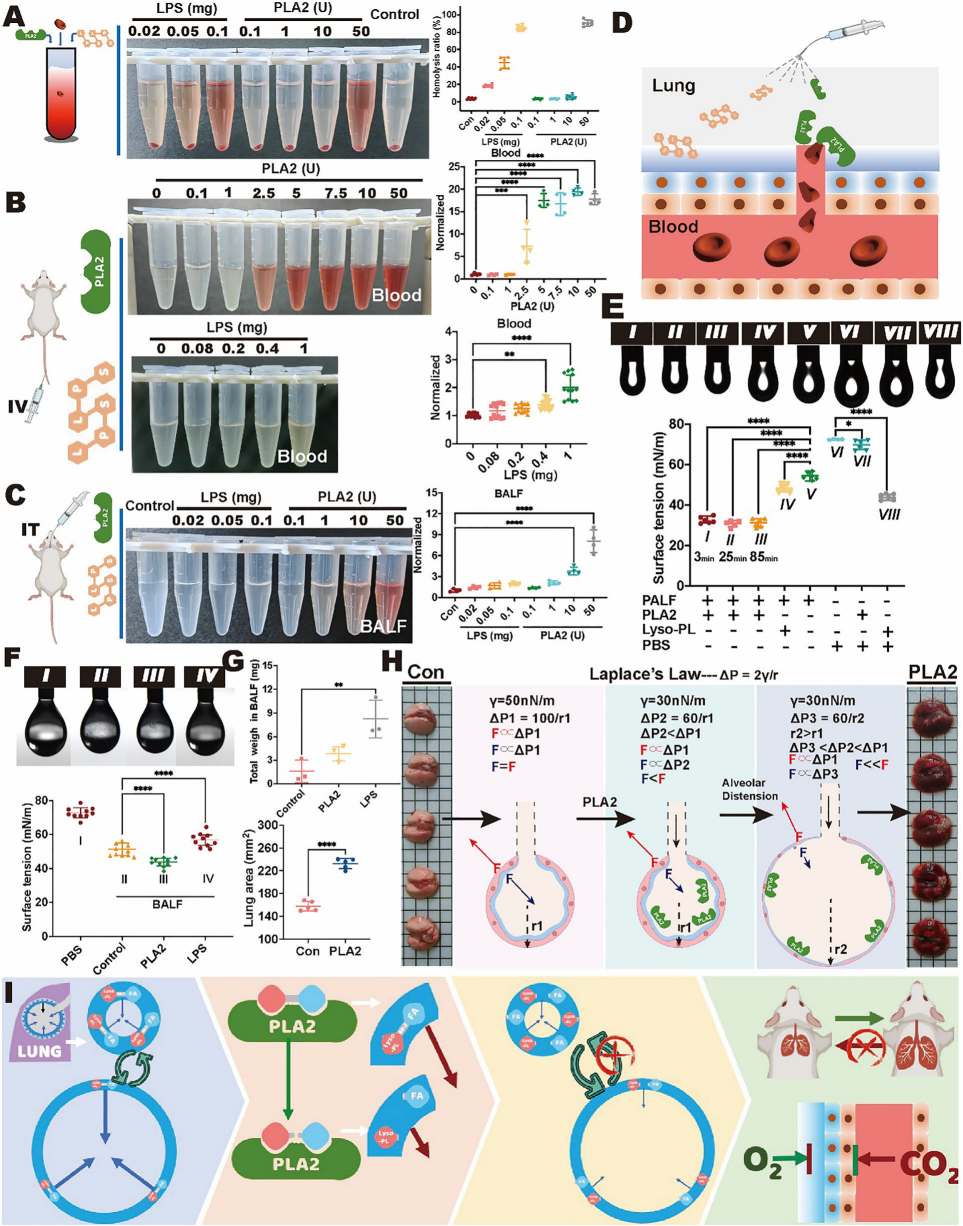
PLA2‐induced hemolysis and pulmonary surface tension alterations. (A) Erythrocyte hemolysis with LPS (0.02–0.1 mg) and PLA2 (0.1–50 U) treatment (*n* = 6). (B) Hemolysis in IV‐administered PLA2 (top) and LPS (bottom) mice blood (PLA2, *n* = 4; LPS, *n* = 12; ^***^ *p* < 0.001, ^****^ *p* < 0.0001). (C) BALF hemolysis after IT‐administered PLA2/LPS (*n* = 4, ^****^ *p* < 0.0001). (D) Schematic of PLA2/LPS disrupting lung barrier integrity via hemolysis. (E,F) Droplet images/surface tension of BALF mixed with PLA2, lyso‐PLs vs. PBS control or collected from mice post PLA2/LPS treatment (E, PBS, *n* = 4; Other groups, *n* = 6. F, *n* = 10; ^*^ *p* < 0.05, ^****^ *p* < 0.0001). (G) Total BALF weight after PLA2/LPS exposure (*n* = 3, ^**^ *p* < 0.01, ^****^ *p* < 0.0001). (H) Mechanism linking PLA2‐induced surfactant degradation to acute respiratory failure (Lung area (left) and morphology (right) following PLA2 IT administration) (*n* = 5). (I) Mechanism schematic of PLA2‐induced acute asphyxiation.

PLA2 and Lipopolysaccharide (LPS) both caused hemolysis in vitro (50U vs. 0.1 mg; Figure 5A) and following intravenous administration in vivo (2.5U vs. 1 mg IV; Figure 5B). However, a critical difference emerged via IT administration: while LPS had no acute effect, PLA2 (50 U) induced immediate erythrocyte lysis within the circulation, and the resulting lysed erythrocytes were then penetrated and detected in BALF (Figure 5C), providing direct evidence that PLA2 disrupts the pulmonary vascular barrier, leading to acute vascular leakage, whereas LPS primarily triggers a systemic inflammatory response but without causing immediate barrier failure (Figure 5D). Collectively, these findings indicate that the acute lethality of PLA2 likely stems from its destruction of the pulmonary barrier, rather than from a generalized inflammatory response. Pulmonary surfactant PLs critically regulate alveolar surface tension, maintain the structural stability of alveolar volume and their associated capillaries [34, 35]. PLA2‐mediated PL degradation sharply reduced BALF surface tension by 40% within 3 min and persisted for 85 min (Figure 5E) when PLA2 was mixed with BALF in vitro. Consistent with this data, in vivo IT administration of PLA2 also decreased BALF surface tension, whereas LPS increased it (Figure 5F), paralleled by changes in BALF weight post‐PLA2 administration (Figure 5G) and aligns with their distinct pathological profiles reported in the literature [36]. According to the Laplace law (P = 2γ/r) [37], this critical loss of surface tension (γ) resulted in a precipitous drop in alveolar retraction pressure (P). This loss of retractive force prevented normal alveolar recoil, leading to overwhelming alveolar overdistension driven by the unchecked outward pull of the chest wall (Figure 5H). Consequently, PLA2‐treated lungs showed 43% volume increase (Figure 5H left) due to the alveoli insufficient recoil force from critically low surface tension, leading to acute asphyxiating death as illustrated in Figure 5I.

### 2.3 Role of PLA2 in Severe Pulmonary Diseases

#### 2.3.1 PLA2‐Induced Disruption of Pulmonary Barrier Function

Evans blue (EB) assays revealed distinct PLA2‐induced barrier disruption patterns (Figure S8A–R). Following IT administration, PLA2 severely damaged the alveolar surfactant layer, which enabled extensive EB penetration from the alveolar space into the lung parenchyma (Figure S8A–C). Notably, this damage was characterized by minimal capillary leakage (Figure S8D–F), indicating that the primary defect occurred at the alveolar epithelium. IV‐administered PLA2 resulted in no significant lung injury and EB penetration (Figure S8G–L). As a further control, nerve agents, while causing rapid lethality, were shown to preserve pulmonary barrier integrity (Figure S9). Unlike LPS‐induced bidirectional barrier disruption (Figure S8M–R), PLA2 selectively targeted alveolar exteriors, creating unidirectional permeability from the alveolar space into the interstitium while preserving the function of the capillary endothelium.

#### 2.3.2 Inflammation Promoted PLA2 Permeation into Lungs

**FIGURE 6:**
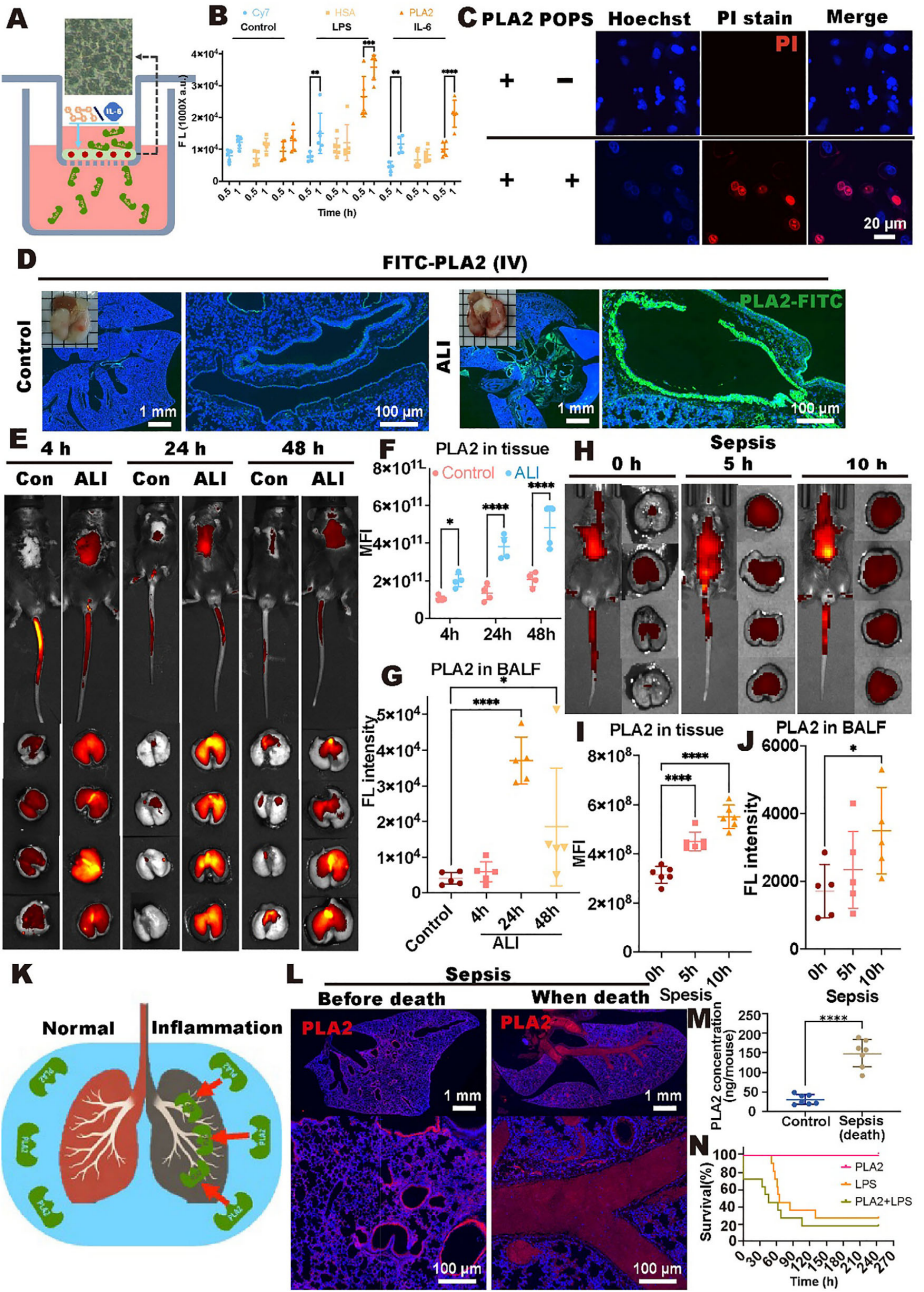
Inflammation promotes PLA2 permeation. (A) Transwell pulmonary barrier model schematic (inset: type II alveolar cell image). (B) Cy7 (cyanine 7, light blue), HSA (yellow) and PLA2 (orange) penetration FI under LPS/IL‐6 stimulation (*n* = 5, ^**^ *p* < 0.01, ^****^ *p* < 0.0001). (C) PI‐stained membrane permeability with POPS+PLA2 (collected from the lower chamber) treatment. (D) Lung fluorescence histopathology (blue: 4′,6‑Diamidino‑2‑phenylindole; green: FITC‐PLA2) and gross images (inset) post‐IV PLA2 in ALI. (E–G) In vivo Cy7‐PLA2 tracking (E), lung/BALF MFI (F,G) in ALI over 48 h (E and F, *n* = 4; G, *n* = 5; ^*^ *p* < 0.05, ^****^ *p* < 0.0001). (H–J) Cy7‐PLA2 tracking (H), lung/BALF MFI (I,J) in sepsis over 10 h (H and I, *n* = 6; J, *n* = 5; ^*^ *p* < 0.05, ^****^ *p* < 0.0001). (K) PLA2 penetration: normal vs. inflamed lungs. (L) Pre‐/post‐mortem PLA2 immunofluorescence in septic lungs. (M) Pulmonary PLA2 levels in septic mice at death (*n* = 7, ^****^ *p* < 0.0001). (N) Survival rates for PLA2, LPS, and PLA2+LPS treatments. FI, Fluorescence intensity; MFI, mean fluorescence intensity; POPS, 1‐palmitoyl‐2‐oleoylphosphatidylserine; HSA, human serum albumin (*n* = 10).

Inflammation‐mediated PLA2 pulmonary penetration may represent a key pathogenic mechanism in severe pulmonary diseases. In transwell models, inflammatory stimuli (LPS/IL‐6) disrupted pulmonary barrier integrity (Figure 6A,B), which permitted the penetration of PLA2—but not Human Serum Albumin (HSA)—while preserving PLA2's catalytic activity (Figure 6C). Notably, direct pulmonary administration of single inflammatory cytokines such as IL‐6 or TNF‐α induced almost no barrier disruption (Figure S10) or pathological effects (Figure 1A), highlighting the importance of a more complex inflammatory milieu, such as that provided by LPS, for significant barrier compromise. Consistently in vivo, fluorescently labeled PLA2 accumulated in lungs during LPS‐induced pneumonia (Figure 6D–G), as well as in a septic model (Figure 6H–J). This inflammation‐dependent permeation was not observed in nerve agent‐exposed controls, despite similar acute lethality (Figure S11). These findings indicate that pulmonary inflammation directly mediated PLA2 permeation into alveoli through barrier disruption (Figure 6K), while selectively excluding proteins such as HSA.

The role of PLA2 translocation from the blood to the alveoli in inducing mortality was further validated. Notably, although PLA2 levels in septic lungs were already significantly elevated compared to controls in surviving subjects (Figure 6H), an exceptionally high spike of PLA2 was detected throughout the lungs and trachea (Figure 6L) immediately after death. Quantitatively, the PLA2 concentration in lung tissue exhibited a dramatic surge to 151 ng, a sharp increase from the pre‐mortem level of 29 ng (Figure 6M). To directly test the hypothesis that circulating PLA2 becomes lethal only upon pulmonary barrier disruption, a combination of LPS (IT) and PLA2 (IV) was administered. The results demonstrated that this combination caused acute lethality, whereas neither agent alone at the same doses induced rapid mortality (Figure 6N). These findings establish that circulating PLA2 can trigger rapid death when the pulmonary barrier is compromised.

#### 2.3.3 Impact of Aging on PLA2 Activity and Pulmonary Barrier Function

**FIGURE 7:**
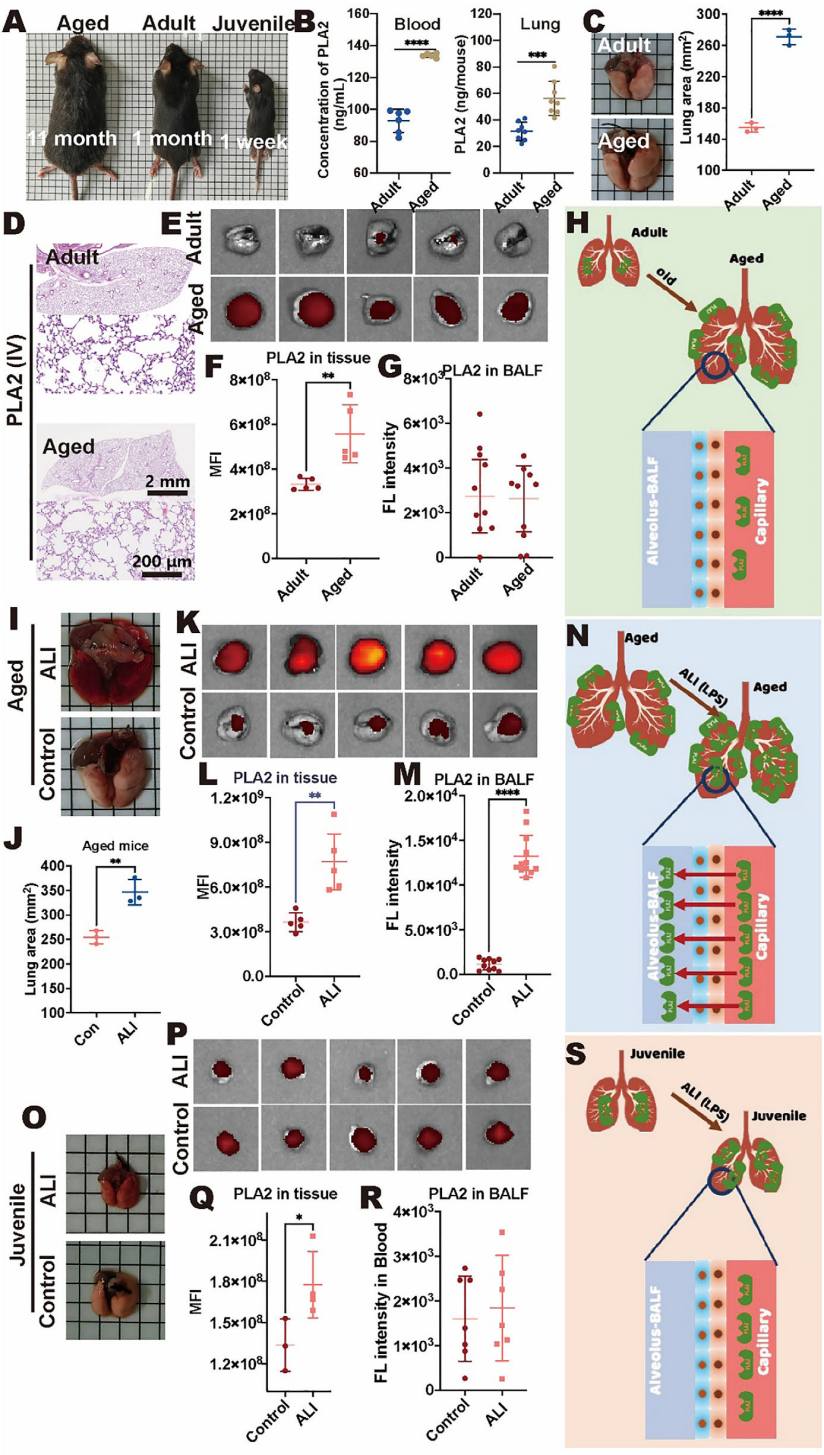
Impact of aging. (A) Mouse image of different ages. (B) The expression of PLA2 in adult and aged mice (*n* = 8, ^***^ *p* < 0.001, ^****^ *p* < 0.0001). (C) Photographs (left) and lung area measurements (right) across age groups (*n* = 3, ^****^ *p* < 0.0001). (D) H&E‐stained lung pathology in adult/aged mice after low‐dose IV PLA2. (E–G) In vivo Cy7‐PLA2 tracking (E) with lung (F)/BALF (G) MFI in adult/aged mice (E and F, *n* = 5; G, *n* = 10; ^**^ *p* < 0.01). (H) Schematic of age‐dependent PLA2 accumulation (alveolar exclusion in aged mice). (I,J) Lung morphology (I) and area quantification (J) in aged mice pre‐/post‐ALI (J, *n* = 3, ^**^ *p* < 0.01). (K–M) Cy7‐PLA2 dynamics (J,K) with lung (L,M)/BALF (K,L) MFI in ALI‐aged mice (K and L, *n* = 5. M, control, *n* = 10; ALI, *n* = 12; ^**^ *p* < 0.01, ^****^ *p* < 0.0001). (N) Schematic of enhanced PLA2 permeation in injured aged lungs. (O) Juvenile mouse lungs pre‐/post‐ALI. P‐R) Cy7‐PLA2 distribution (P) with lung (P,Q)/BALF (P,R) MFI in ALI‐juvenile mice (P, *n* = 4; Q, control *n* = 3, ALI *n* = 4; R, *n* = 7; ^*^ *p* < 0.05). (S) Schematic of juvenile lung resistance to PLA2 permeation during inflammation.

Sepsis and severe pulmonary diseases are associated with elevated mortality in the elderly [38, 39], warranting investigation of interactions between aging, PLA2, and barrier function (Figure 7A). Under healthy conditions, aged mice exhibited ∼ 44% higher blood PLA2 levels (131–135 ng/mL) compared to adults (82–99 ng/mL) and a near two‐fold higher pulmonary PLA2 (56 ng/lung) compared to adults (31 ng/lung, Figure 7B). Consistent with known age‐related physiological changes, aged mice exhibited 68% greater lung area (Figure 7C) and showed increased PLA2 retention in lungs/heart/kidneys/brain (Figure 7D–F; Figure S12) compared to adults, although no PLA2 was detected in BALF (Figure 7G). This suggests that the aged lung is at a heightened risk for PLA2 infiltration and accumulation (Figure 7H).

During LPS‐induced ALI, aged mice developed more severe pathology, exhibiting pulmonary hemorrhage (Figure 7I), 40% lung expansion (Figure 7J), and increased pulmonary PLA2 retention (Figure 7K,L). Critically, PLA2 in blood quickly raised to 143 ng/mL within 4 h (Figure S13A). At the same time, BALF PLA2 levels in aged mice with ALI surged 13‐fold (Figure 7M), a significantly greater increase than the 9‐fold rise observed in ALI‐affected adults (Figure 6G). PLA2 predominantly accumulated in lungs/heart/kidneys but no brains as before (Figure S14), further supporting the stability of the blood–brain barrier under these conditions. Consequently, ALI mortality rises markedly, reaching 43% within 4 h in aged mice, whereas under the same conditions, young adult mice show 0% mortality (Figure S13B). In summary, aged mice exhibit inherent pulmonary PLA2 accumulation, and the enhanced inflammatory permeation during ALI accelerates mortality compared to adults (Figure 7N).

Juvenile mice showed minimal PLA2 translocation despite LPS exposure, presenting only minor pulmonary hemorrhage (Figure 7O) and limited accumulation in the lungs and liver (Figure 7P–Q; Figure S15), with no PLA2 increase detected in the BALF (Figure 7R). These results (Figure 7S) demonstrate that juvenile mice possess more robust pulmonary barriers that effectively resist inflammation‐induced PLA2 translocation. Collectively, age‐dependent barrier dysfunction exacerbates both PLA2 accumulation/leakage and mortality risk.

#### 2.3.4 Association Between PLA2 and Severe Pulmonary Diseases

Clinical data suggest a potential link between PLA2 and rapidly fatal pulmonary diseases (Figure S16A). When healthy, PLA2 remains stable in the body. Even with exogenous intravenous administration, plasma PLA2 rises only slightly to ∼115 ng/mL and persists at this level for up to 5 days (Figure S17). Existing research in critical illness has established that PLA2, while essential in antimicrobial defense, becomes a key mediator of systemic inflammation and organ injury when dysregulated and was upregulated via NF‐κB [40], surfactant hydrolysis [41], pro‐inflammatory effects [42], and association with poor outcomes in severe pulmonary diseases. In these experimental models, sepsis (Figure S16B) and acute lung injury (ALI; Figure S16D) were characterized by sharply increased PLA2 levels (Figure S16C,E) and a >70% 24 h mortality rate (Figures S16 and S18), which correlated with severe pulmonary pathology including hemorrhage and edema. Literature reports indicate serum PLA2 concentrations of approximately 284 ng/mL in septic patients [43], which aligns closely with the measurement of 276 ng/mL in septic mice (Figure S16C). In contrast, neonatal hypoxia (Figure S16F) presented with pulmonary hemorrhage in the absence of PLA2 elevation (Figure S16G), a discrepancy that may be attributed to technical limitations or distinct pathophysiology. Taken together, these findings strongly implicate PLA2 dysregulation in the pathogenesis of acute pulmonary fatalities, with neonatal hypoxia representing a notable exception.

As demonstrated in models, the septic state triggers extensive translocation of PLA2 from the systemic circulation into lung tissue (Figure 5D), culminating in significant pulmonary accumulation (Figure 6H–J). Quantitatively, while pulmonary PLA2 in healthy mice remains at ∼29 ng, it rises to ∼104 ng (Figure S16C) in septic mice and further escalates to 151 ng at the point of mortality (Figure 6M). Critically, this accumulated level of 104 ng approximates the exogenous lethal dose of 126 ng (0.075 U) established via IT administration (Figure 1E). In healthy mice, blood contains approximately 97–116 ng of PLA2 (Figure S16B; 97.49 ng/mL; estimated blood volume: ∼1.2 mL). Thus, even if all PLA2 from the blood of a healthy mouse entered the lungs, it would only approach the lethal threshold. These findings demonstrate that endogenously accumulated PLA2 during sepsis not only reaches but can exceed the acutely lethal threshold, confirming its role not as a passive biomarker but as an active executor of terminal organ failure. Importantly, this mechanism requires the convergence of two preconditions grounded in established theory [37]: inflammation‐induced systemic upregulation of PLA2 and inflammation‐induced breakdown of the pulmonary alveolar‐vascular barrier [42, 44]. Furthermore, the direct cause of death is also explained by established principles, such as Laplace's law in the context of alveolar overdistension following surfactant degradation (Figure 5) [37].

In summary, this work characterizes PLA2 as an endogenous “time bomb.”(Figure S19) Its physiological presence (29–31 ng in lung tissue; 85–110 ng/mL in plasma, as shown in Figure S16) represents the “stable explosive,” even remaining non‐lethal even at systemic doses of 1–50 U in blood, without causing mortality (Figure 2D), hemolysis (Figure 5B), or pulmonary impairment (Figure 2Ciii). The pulmonary barrier functions as the “safety pin” by segregating circulating PLA2 from alveolar surfactant (Figure 3B). Inflammatory barrier disruption acts as the “ignition switch,” while the duration of barrier integrity serves as the “timer.” Once activated, the upregulated PLA2 rapidly penetrates into the alveoli, degrades surfactant PLs, eliminates surface tension, and causes fatal asphyxia, thereby completing the mechanistic analogy as illustrated in Figure S19.

### 2.4 Treatment of PLA2‐Induced Fatal Diseases

#### 2.4.1 Screening of Target PLs and PLA2 Inhibitor

**FIGURE 8:**
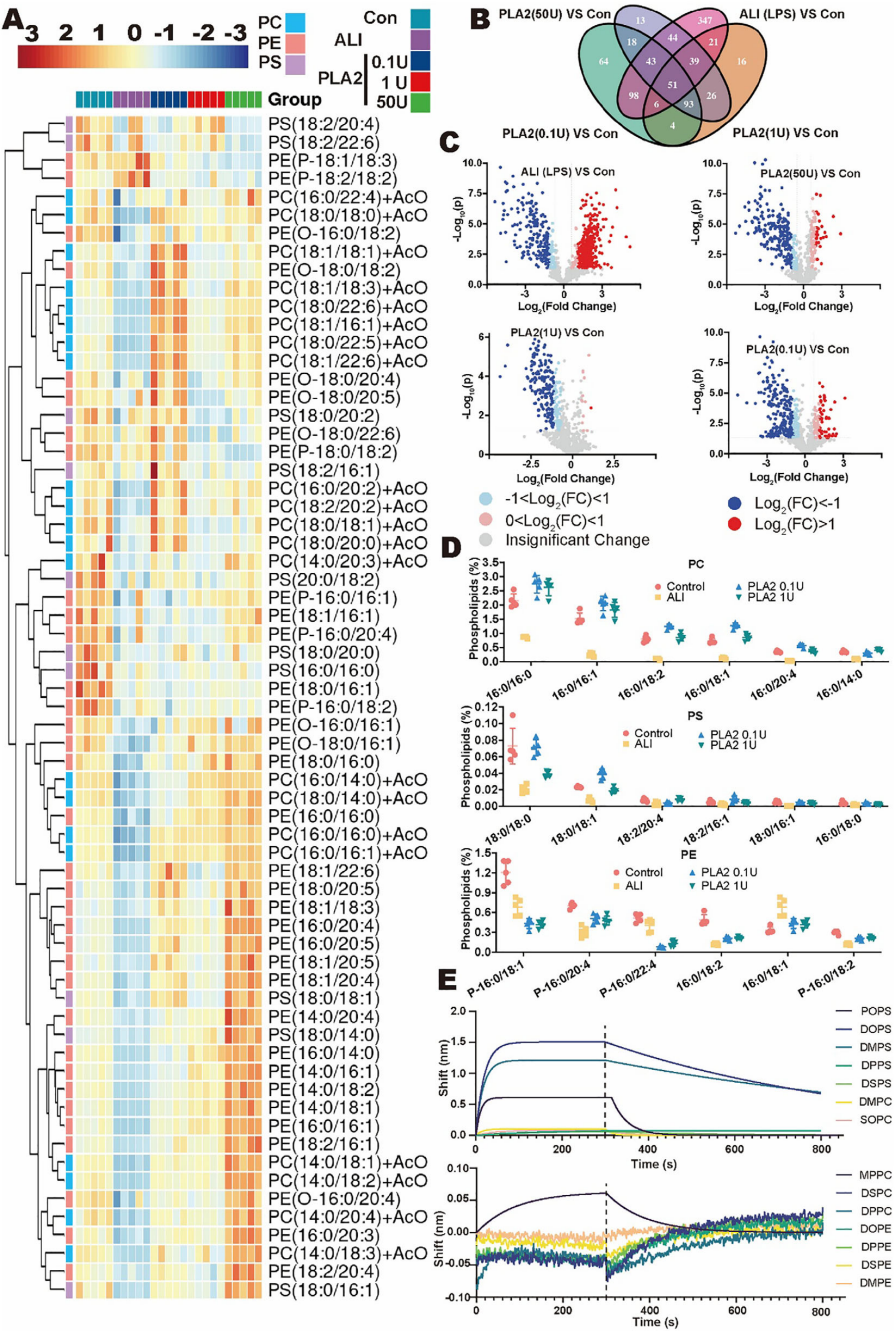
Screening of target PLs and PLA2 inhibitor. (A) Heatmap of BALF PL profiles in ALI/PLA2‐exposed mice (*n* = 5). (B) Venn diagram of 887 overlapping BALF PLs across experimental groups (*n* = 5). (C) Volcano plot of differentially expressed BALF PLs (*n* = 5). (D) Abundance scatter plots of the top six BALF PLs (*n* = 5). (E) Wavelength shifts during PL‐PLA2 binding/dissociation (dotted line separates phases).

PLs exhibit therapeutic potential by inhibiting PLA2‐mediated degradation. Lipidomics revealed 66/184 BALF PL species altered post‐injury (Figure 8A; Figure S20). PLA2 induced significantly fewer PL alterations than LPS across pulmonary injury models (Figure 8B). Specifically, PLA2 downregulated 50% PE and 42% PS species (Figure 8A,C) and further analysis of dominant PLs confirmed PLA2 preferentially targets PE/PS (Figure 8D; Table S2), suggesting their therapeutic potential as competitive PLA2 inhibitors.

High‐affinity PL‐PLA2 binding enhanced enzymatic sequestration and inhibited hydrolysis. PS demonstrated superior PLA2‐binding affinity vs. PC/PE (Figure 8E), explaining why weakly‐bound DPPC (Figure 8E) caused minimal injury (3D, Figure S7) despite its abundance (Figures 3C and 8D). Among the PLs tested, DOPS and POPS exhibited the highest binding rates to PLA2 (Table S3). An important consideration in therapeutic design is that the lyso‐PS (16:0) generated from POPS hydrolysis exhibits inherent tissue irritancy and cytotoxicity (Figure 4E,F; Figure S7). Therefore, integrating the structure‐activity data of dioleoyl groups (Figures 3D–F and 8E) identified DOPS as it leverages the safety profile of the dioleoyl moiety and the high binding efficacy of the PS.

The Phase III VISTA‐16 trial of varespladib in acute coronary syndrome was terminated early, an outcome attributed to its broad inhibition of both pathogenic and potentially protective PLA2 isoforms [45]. Despite this setback in the cardiovascular domain, varespladib was prioritized for sepsis intervention based on its established clinical safety profile from prior studies. To date, varespladib and its prodrug remain the only PLA2 inhibitors advanced to clinical stages for non‐envenomation indications [46]. This stands in contrast to the numerous anti‐inflammatory agents—such as TNF, IL‐1, and IL‐6 antagonists—that have entered clinical trials for sepsis yet failed to improve outcomes, underscoring the challenge of targeting purely inflammatory pathways in critical illness [45, 47].

#### 2.4.2 Treatment of PLA2‐Induced Acute Pulmonary Toxicity and Mortality

**FIGURE 9:**
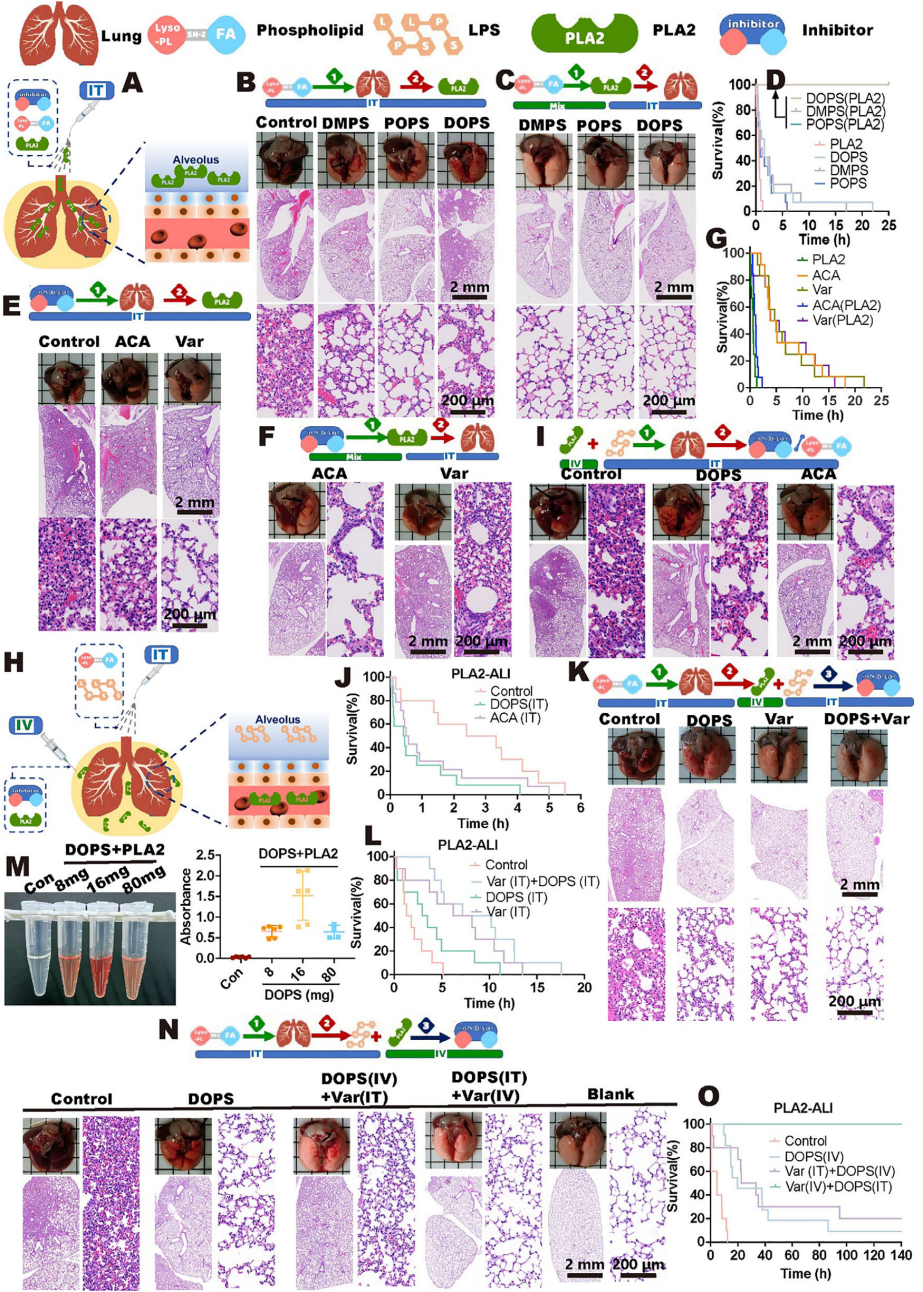
Treatment of PLA2‐induced fatal pulmonary diseases. (A) Schematic of PLA2 exposure model with PL/inhibitor treatments. (B–D) Therapeutic effects of prophylactic PLs (B) vs. pre‐mixed PLs+PLA2 (C): schematics (top), lung photographs (middle), H&E histopathology (bottom), and survival (D) (D, *n* = 10). (E–G) Prophylactic inhibitor (E) vs. pre‐mixed inhibitor+PLA2 (F) effects: schematics (top), lung photographs (middle), H&E histopathology (bottom), and survival (G) (G, *n* = 10). (H) Schematic of PLA2‐enhanced ALI model with PL/inhibitor interventions. (I–L) Therapeutic effects in ALI mice with DOPS/ACA (I,J) or varespladib (K,L): schematics (top), lung photographs (middle), H&E histopathology (bottom), and survival (J,L) (J and L, *n* = 10). (M) Hemolysis assay of DOPS+PLA2 mixtures (M, DOPS 80 mg group, *n* = 4; other groups, *n* = 6). (N,O) Combination therapy (DOPS+varespladib) effects via different administration routes: schematics (top), lung photographs (middle), H&E histopathology (bottom), and survival (O). DOPS, dioleoylphosphatidylserine; ACA, anthranilic acid; Var, varespladib (survival curve, *n* = 10).

PLA2 directly targeted alveolar PLs to mediate acute lethality (Figure 9A). PL administration timing critically determined its protective efficacy: prophylactic administration of POPS/DMPS/DOPS reduced pulmonary injury (Figure 9B) and extended survival to 7–22 h (vs. 1 h control). Strikingly, pre‐mixing these PLs with PLA2 prior to administration completely prevented lethality (Figure 9C,D). indicated that preemptive sequestration of PLA2 is essential for optimal protection, with DOPS demonstrating superior barrier‐stabilizing capacity. In contrast, PLA2 inhibitors—including the broad‐spectrum Arachidonyl Trifluoromethyl Ketone (ACA) and the type II‐specific varespladib, exhibited different time‐dependent efficacy: prophylactic administration was more effective than pre‐mixing (Figure 9E–G). Among the inhibitors tested, varespladib demonstrated the highest therapeutic potency against acute lethality.

#### 2.4.3 Treatment of PLA2‐Enhanced ALI

To model the pathological scenario in which circulating PLA2 penetrates an inflammation‐weakened pulmonary barrier, a PLA2‐enhanced ALI model was established (Figures 9H and 8M) by LPS (IT) plus PLA2 (IV) administration. Consistent with their mechanisms of action, post‐exposure administration of either ACA or DOPS failed to confer protection (Figure 9I,J), A combination of prophylactic DOPS plus therapeutic varespladib significantly improved survival outcomes (Figure 9K,L), demonstrating distinct administration requirements for PLs vs. inhibitors.

The optimized combination therapy was further explored. DOPS was administered via the IT route to avoid the hemolysis observed when it meets PLA2 in the bloodstream upon IV injection (Figures 9M and 3F; Figure S21), while varespladib was delivered IV to optimally inhibit circulating PLA2. The optimized combination therapy eliminated hemorrhage (Figure 9N) and achieved 133 h survival, a dramatic improvement over the 2 h survival in controls (Figure 9O). The regimen not only surpassed the efficacy of all monotherapies but also nearly completely rescued even severe inflammatory lung injury.

#### 2.4.4 PLA2 Antagonist Therapy for ALI and Sepsis

**FIGURE 10:**
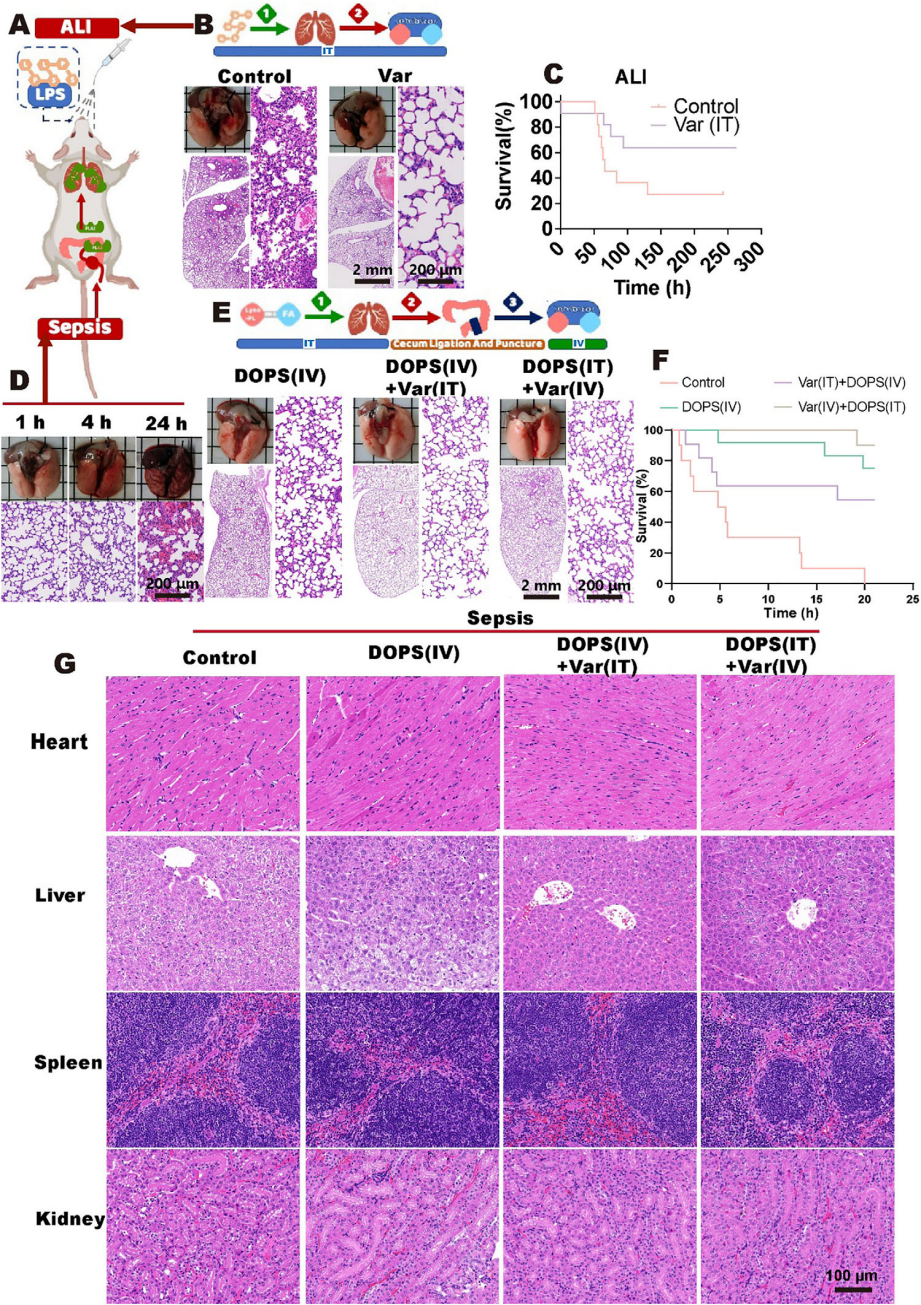
Therapy for ALI and Sepsis. (A) Schematic of sepsis and ALI mouse models. (B,C) Varespladib treatment in ALI: schematic (B, top), lung images (B, left), H&E sections (B, bottom/right), and survival (C, *n* = 10). (D) Sepsis progression: lung photographs (top) and H&E sections at different timepoints. (E–G) Varespladib and DOPS efficacy in sepsis: schematic (E, top), lung images (E, left), H&E sections (E, bottom/right), survival (F, *n* = 10), and other organ histology (G).

The efficacy of the optimized therapeutic regimen for ALI and sepsis was evaluated as outlined in Figure 10A. To establish the ALI model, aerosolized LPS was employed to activate the TLR4 pathway [48], triggering a robust inflammatory cascade characterized by neutrophil recruitment, release of destructive effectors (elastase, reactive oxygen species), and increased vascular permeability, which collectively lead to alveolar‐capillary barrier damage and permeability‐type pulmonary edema. In ALI model, varespladib alone reduced lung hemorrhage and mortality (70%→30%; Figure 10B,C); in sepsis model (Figure 10D), combination therapy (IV varespladib + pro‐IT DOPS) preserved lung integrity (Figure 10E) and achieved 100% 24 h survival, a stark contrast to the 100% 24 h mortality observed in untreated controls (Figure 10F), with nearly 70% survival even at 50 h. Notably, all treatment regimens (IT DOPS, IV varespladib, and their combination) demonstrated a favorable safety profile, as evidenced by the absence of significant damage in major organs (Figure 10G; Figure S21). Mechanistically, IV varespladib inhibited circulating PLA2, reducing lung barrier damage, while IT DOPS neutralized alveolar PLA2, synergistically protecting surfactant, and barrier function. This dual approach significantly enhanced survival in severe pulmonary disease and sepsis.

## 3 Conclusion

This study demonstrates that PLA2 exhibits a unique dual‐toxicity profile, combining rapid chemical toxin‐like lethality (“electric shock‐like” death) with extreme biological toxin‐level toxicity. As an endogenous enzyme, PLA2 remains physiologically dormant but transforms into a potentially lethal effector upon pathological activation. Pulmonary PLA2 accumulation rapidly degrades surfactant PLs, causing >30% reduction in alveolar surface tension that precipitates alveolar collapse, acute respiratory failure, and fatal asphyxiation. These findings not only elucidate PLA2's rapid lethality mechanism but also provide a pathophysiological basis for acute mortality in late‐stage sepsis and ALI.

Further investigations reveal that disease‐associated inflammation both upregulates PLA2 expression and compromises pulmonary barrier integrity, permitting PLA2 transmigration and subsequent lethal activation. Moreover, age‐related deterioration of barrier function further exacerbates PLA2‐mediated pathogenicity in respiratory diseases, underscoring the critical importance of barrier preservation as a therapeutic strategy. Notably, conventional interventions (e.g., anti‐inflammatory agents/mechanical ventilation) prove ineffective once PLA2 activation has occurred, due to the ensuing irreversible alveolar overdistension and lung overinflation. To address this therapeutic challenge, a dual‐targeting strategy was developed: (1) IT administration of DOPS for direct PLA2 neutralization in alveoli, and (2) IV administration of varespladib for systemic PLA2 inhibition. This optimized regimen increased survival rates from 0% to over ≥90% in PLA2 intoxication, sepsis, and ALI models. These results establish PLA2 as a central executor in critical pulmonary pathologies and introduce a novel alveolar‐protective therapeutic paradigm with significant clinical translation potential.

## 4 Materials and Methods

### 4.1 Cells and Animals

Lewis Lung Carcinoma (LLC) Cells were cultured in Dulbecco's modified Eagle's medium (DMEM) supplemented with 10% fetal bovine serum (FBS), penicillin (100 U/mL), and streptomycin (100 mg/mL) at 37°C ± 0.5°C with 5% CO_2_ and 90% relative humidity, and passaged every 2–3 days. Alveolar Type II (AT2) Cells were cultured in medium of DMEM/F12 (1:1), while all other culture conditions remained consistent with those used for LLC Cells.

All experiments were conducted in accordance with the “Regulations for the Administration of Laboratory Animals” issued by the National Science and Technology Commission of the People's Republic of China (November 14, 1988). The study was performed following the ARRIVE guidelines and the experimental animal management and use guidelines of the Beijing Institute of Pharmacology and Toxicology, with approval from the Beijing Animal Ethics Committee (IACUC permit number: IACUC‐DWZX‐2025‐565). Juvenile C57BL/6N mice (1‐week‐old, 4.5 ± 0.5 g, mixed sex), adult C57BL/6N mice (6‐week‐old, 20 ± 2 g, male), and aged C57BL/6N mice (11‐month‐old, 28 ± 4 g, male) were purchased from Beijing Vital River Laboratory Animal Technology Co., Ltd. Rodents were provided with ad libitum access to sterile food and distilled water. Animals were housed in stainless steel cages with wood shaving bedding under controlled environmental conditions (12:12 h light/dark cycle) in air‐conditioned rooms. Animals were anesthetized by intraperitoneal injection of sodium pentobarbital to ensure surgical‐level anesthesia. Depth of anesthesia was confirmed by the absence of pedal withdrawal reflexes. All procedures were performed under aseptic conditions to minimize pain and distress.

### 4.2 Animal Models

#### 4.2.1 ALI

The ALI model was established via direct IT LPS installation. The pulmonary liquid quantitative atomizing needle was rinsed with distilled water prior to use. Briefly, the atomizing needle was inserted into the trachea through the oropharynx under laryngoscopic guidance in the supine position of the anesthetized mice. LPS (50 µL, 1 mg/mL) was administered by atomizing needle within 3 s to ensure optimal aerosol droplet formation. Following administration, animals were immediately positioned in lateral recumbency to maintain airway patency and facilitate recovery [49].

#### 4.2.2 Sepsis

The sepsis model was established using the classic cecal ligation and puncture (CLP) protocol as previously described in studying systemic inflammatory responses. Given the irreversible nature of the injury from CLP, non‐treatment group animals were humanely euthanized at 24 h post‐CLP, while treatment group animals were humanely euthanized at 72 h post‐CLP [50].

### 4.3 Survival Rate Assessment

In the inflammatory biomarker exposure study, a panel of inflammatory mediators—including IL‐6, TNF‐α, INF‐γ, CRP, MCP‐1, COX‐2, and MMP‐9—was administered to C57BL/6N mice via IT nebulization. Under anesthesia, a 50 µL solution containing a 0.42 µg bolus of each respective biomarker was delivered directly into the lungs using a specialized tracheal nebulization needle under laryngoscopic guidance. In a separate PLA2 exposure study, the same nebulization procedure was employed to administer PLA2 at concentrations of 0.05, 0.075, 0.1, 0.25, 0.5, 5, and 50 U. Following all administrations, mice were placed in lateral recumbency to maintain airway patency.

### 4.4 Preparation of Liposomes

All PL were prepared in the form of liposomes for investigation. Liposomes (DMPC, DPPC, MPPC, SOPC, DMPE, DPPE, DSPE, DOPE, DMPS, DPPS, DOPS, DSPS and POPS) were prepared by the thin‐film dispersion method [51]. Briefly, 8 mg of PL was dissolved in chloroform and evaporated under reduced pressure to form a thin lipid film. Subsequently, the film was then hydrated with 1 mL of PBS. The mixture was sequentially sonicated using an ultrasonic disruptor (30% amplitude, 5 s on/5 s off cycles for 2 min) to form a homogeneous liposomal suspension. The liposomes were stored at 4°C for further use.

### 4.5 In Vitro Cell Viability

Cell Counting Kit‐8 (CCK‐8) assay was employed to assess the viability of LLC cells treated with PL+PLA2 mixture. Cells (1 × 10^5^/well) were seeded in 96‐well plates for 24 h to allow attachment. Twelve PLs (0.8 mg/mL)—representing phosphatidylcholines (DMPC, DPPC, MPPC, and SOPC), phosphatidylethanolamines (DMPE, DPPE, DSPE, and DOPE), and phosphatidylserines (DMPS, DPPS, DSPS, and DOPS) were individually mixed with PLA2 at three enzyme concentrations (1, 0.1, and 0.01 U/well). The mixtures were then added into wells for 10 min at 37°C. After treatment, the cells were incubated with CCK‐8 reagent for 2 h at 37°C, and absorbance was measured at 450 nm using a microplate reader (*n* = 6 replicates per condition).

### 4.6 Confocal Microscopy for PI Staining

To assess the activation environment of PLA2, LLC cells were incubated with the enzyme of PLA2 in the presence of various biological fluids, including phosphate‐buffered saline (PBS), serially diluted serum (100×, 10×, and 1×), diluted erythrocyte debris (100×, 10×, and 1×), and BALF. Following incubation, cellular membrane integrity was assessed by PI staining and visualized using laser scanning confocal microscopy (LSCM).

To investigate the cellular effects of PL‐PLA2 interactions, cells were treated with various PLs (0.8 mg/mL, DMPC, DPPC, MPPC, SOPC, DMPE, DPPE, DSPE, DOPE, DMPS, DPPS, DSPS, and DOPS) mixed with PLA2 (0.01, 0.1, and 1 U/mL) for 10 min, respectively, and then stained with PI (50 µg/mL), imaging by LSCM with Hoechst 33342 staining.

To further identify the specific cytotoxic components, the dose‐dependent cellular damage induced by lysophospholipids (lyso‐PLs) was systematically evaluated. LLC cells were incubated at 37°C for 10 min with various concentrations of lyso‐PLs: 5, 50, and 500 µg of Lyso‐PE, Lyso‐PS (18:1), and Lyso‐PC, as well as 0.05, 0.5, and 5 µg of Lyso‐PS (16:0). Following incubation, cell membrane integrity was assessed by PI staining and visualized using LSCM.

### 4.7 Penetration Studies Using the Transwell System in Vitro

#### 4.7.1 Establishment of the Transwell System with Lung Barrier

AT2 cells were seeded on a 0.4 µm pore Transwell insert (24 mm Transwell, Corning) at a density of 1 × 10^5^ cells/insert and cultured in Gibco DMEM/F12 (1:1) medium for 1 week to form a confluent monolayer. Monolayer integrity was confirmed by performing a permeability assay after 4 h of culture [52], after which subsequent experiments could proceed.

#### 4.7.2 Evaluation of Pulmonary Barrier Integrity Under Inflammation

The pulmonary barrier model was divided into three experimental groups: (1) control group with 100 µL PBS in the apical chamber; (2) mild inflammation group, treated with 100 µL of 0.2 ng/mL LPS; and (3) severe inflammation group, treated with 100 µL of 0.5 ng/mL recombinant human IL‐6. Following 37°C incubation for 30 min, the stimulating solution was removed, and then 200 µL test solutions of FITC‐labeled PLA2 (0.02 mg/mL), FITC‐labeled HSA (0.02 mg/mL), or Cy7 (0.01 mg/mL), were added to the apical chambers of each treatment group, respectively. 50 µL aliquots were collected from the basolateral chamber at 0.5 and 1 h intervals, diluted 10‐fold with PBS, and subjected to fluorescence quantification to assess barrier permeability dynamics. Fluorescence quantitative analysis was performed using a multifunctional microplate reader. (Cy7: λex = 750 nm/λem = 775 nm), (FITC: λex = 488 nm/λem = 525 nm)

#### 4.7.3 Post‐Penetration Enzymatic Activity Evaluation

Following translocation across the Transwell system under LPS‐stimulated inflammatory conditions, the PLA2 was collected from the basolateral chamber and mixed with 0.8 mg/mL POPS liposomes in an equal volume, and then co‐incubated with LLC cells (1 × 10^6^ cells/mL, 37°C, 5% CO_2_). 10 min later, the LLC cells were stained with Hoechst 33342 and PI, and imaged using LSCM.

### 4.8 Hemolysis Assay

#### 4.8.1 In Vivo

The BALF was collected from the mice 1 h after IT administration of different concentrations of PLA2 (0.1, 1, 2.5, 5, 7.5, 10, and 50 U) or LPS (0.08, 0.2, 0.4, and 1 mg) using atomizing needle and then centrifuged at 3000 × g for 10 min at 4°C to pellet cellular debris and insoluble particulates. The total volume was adjusted to 1 mL for subsequent analysis. The BALF from untreated mice was tested as a negative control. The supernatant absorbance was measured at 550 nm.

#### 4.8.2 In Vitro

For hemolysis evaluation, 0.5 mL of test solutions containing LPS (0.02, 0.05, or 0.1 mg) or PLA2 (0.1, 1, 10, or 50 U) were mixed with 0.5 mL of a 2% erythrocyte suspension in physiological saline, followed by incubation at 37°C for 1 h. After centrifugation (3000 rpm, 10 min), the supernatant absorbance was measured at 550 nm. PBS and distilled water served as the blank control and 100% hemolysis control, respectively. The hemolysis rate (%) was calculated as:

$$\begin{array}{ccc} & & \mathrm{Hemolysis}\;\left(\%\right)\\ & & \;=\left[\left(OD_{\mathrm{test}}-OD_{\mathrm{blank}}\right)/\left(OD_{\mathrm{control}}-OD_{\mathrm{blank}}\right)\right]*100\% \end{array}$$

### 4.9 Affinity Assay Between PLs and PLA2

PLA2 was biotinylated (bioPLA2) at a 1:2 molar ratio (protein: biotin) and immobilized on SMAP probes (∼10 µg/mL). Liposomes were prepared as described before the concentration of 500 µg/mL. Nonspecific binding was assessed with blank probes. Binding/dissociation curves were recorded using a label‐free biomolecular analyzer (Suzhou Xiaoe Biotechnology Co., Ltd.), with slopes calculated automatically.

### 4.10 Lipidomic Analysis of Lipidomics in BALF

BALF was sequentially collected from mice following IT treatment with LPS (1 mg/mL) or PLA2 (0.1, 1, or 50 U), respectively, centrifuged at 3000 × g for 15 min at 4°C to remove cellular debris and large particulates, and the supernatant was concentrated via lyophilization. Lipid analysis was performed using an ultra‐high performance liquid chromatography system (Shimadzu) coupled with a mass spectrometer (AB SCIEX). System stability and method reproducibility were evaluated prior to sample analysis. Targeted lipid profiling was conducted in both positive and negative ion modes, with characteristic ion pairs detected using multiple reaction monitoring. Data acquisition and quantitative analysis were performed using MultiQuant software, and quality control was implemented based on peak area integration integrity, retention time consistency, and standard curve linearity.

### 4.11 Distribution of PLA2 Following Different Administration Routes In Vivo

Adult C57BL/6N mice were administered 0.02 mg of FITC‐labeled PLA2 via intranasal instillation, intravenous injection, inhalation, or intratracheal instillation. 1 h after administration, serum was collected by centrifugation at 3000 rpm for 15 min. Meanwhile, heart, liver, spleen, lungs, and kidneys were collected and homogenized, followed by centrifugation at 5000 × g for 10 min to obtain tissue supernatants. All samples (serum and tissue supernatants) were diluted 10‐fold with PBS, and fluorescence intensity was measured using a multifunctional microplate reader.

### 4.12 Surface Tension Measurement

#### 4.12.1 In Vitro

BALF was collected from healthy mice, centrifuged at 3000 × g for 10 min at 4°C to remove cellular debris and stored at 4°C for further analysis. For surface tension measurements, 10 µL of PLA2 or Lyso‐PL solution was added to 1 mL BALF, yielding final concentrations of 1 U/mL PLA2 and 0.01 mg/mL Lyso‐PL, respectively. After 3 min of mixing, surface tension was immediately measured via the pendant drop method using a contact angle analyzer (Dataphysics Instruments GmbH). Drops were stabilized to maximum volume without detachment, equilibrated for 3 s, and measured under constant humidity and 25°C ambient temperature. For the PLA2‐BALF group, additional measurements were taken at 25 and 85 min post‐mixing. The surface tension of PBS mixed with the same content of PLA2 and Lyso‐PL was also test as control groups, including: (1) PBS alone, (2) BALF alone, and (3) mixtures of PLA2 or Lyso‐PL with PBS at identical concentrations.

#### 4.12.2 In Vivo

Animals were randomly assigned to receive IT administration of PBS (control), LPS (1 mg/mL), or PLA2 (0.5 U) via specialized nebulization needles, respectively. BALF was collected 1 h post‐exposure from the treated mice and centrifuged at 3000 × g for 10 min at 4°C to remove cellular debris. The supernatant was analyzed for surface tension using a contact angle analyzer.

### 4.13 ALI and Sepsis Therapy

#### 4.13.1 Preparation of PLA2 Inhibitors

Varespladib and ACA were first dissolved in dimethyl sulfoxide at a concentration of 10 mg/mL. The stock solutions were then diluted 10‐fold and dispersed in PBS to prepare working solutions, which were stored at 4°C for subsequent use.

#### 4.13.2 Treatment Regimens

The therapeutic efficacy of DOPS liposomes and varespladib, administered via both IT instillation and IV injection, was evaluated across multiple treatment paradigms: (1) prophylactic administration (given 8 h prior to injury), (2) post‐injury therapeutic intervention (initiated at 1 or 4 h after insult), and (3) combination therapy (sequential IT DOPS followed by IV varespladib). Survival rates and pulmonary injury severity were monitored in all treatment groups.

### 4.14 Statistical Analysis

Statistical significance was determined using an unpaired Student's *t*‐test for comparisons between two groups or one‐way ANOVA with Tukey's post‐hoc test for comparisons among multiple groups. Data are expressed as mean ± SD of three independent experiments. A *p* < 0.05 was considered statistically significant. For details on statistical analyses, tests used, size of *n*, definition of significance, and summaries of statistical outputs, see the corresponding figure legend and Results.

## Author Contributions

**Jianyu Wang**: conceptualization, formal analysis, investigation, methodology, validation, writing – original draft. **Zhongxing Xu**: visualization, investigation, methodology, validation. **Lin Wang**: investigation, methodology, visualization. **Xin Sui**: investigation, validation, methodology, project administration. **Yuan Luo**: funding acquisition, methodology, supervision, writing – original draft. **Xiuli Zhao**: project administration, resources, data curation, supervision. **Jun Yang**: formal analysis, funding acquisition, methodology, conceptualization, project administration, resources, validation, visualization, writing – review & editing. **Yongan Wang**: funding acquisition, project administration, resources, writing – review & editing.

## Ethics Statement

This work has received approval for research ethics from the Institutional Animal Care and Use Committee (IACUC) of Animal Laboratory of Laboratory animal center Academy of Military Medical Sciences (Approval No. IACUC‐DWZX‐2025‐P637).

## Conflicts of Interest

The Authors declare no conflicts of interest.

## Supporting information




**Supporting File 1**: advs74961‐sup‐0001‐SuppMat.docx.

**Supporting File 2**: advs74961‐sup‐0002‐VideoS1.mp4.

**Supporting File 3**: advs74961‐sup‐0003‐VideoS2.mp4.

## Data Availability

The data that support the findings of this study are available from the corresponding author upon reasonable request.
