# Supplementary material for: Endogenous “Time Bomb” – Mislocalized Phospholipase A2 as a Critical Mediator of Ultra‐Rapid Mortality in Sepsis and Acute Lung Injury
Source: Adv Sci (Weinh). 2026 Mar 24;13(32):e14915. doi: 10.1002/advs.202514915 (PMC13252650; doi:10.1002/advs.202514915)
Supplement: Supplementary file 1 — Supporting File 1: advs74961‐sup‐0001‐SuppMat.docx. [file ADVS-13-e14915-s003.docx]

Supporting Information

**Endogenous “Time Bomb” -** **Mislocalized Phospholipase A2 as a Critical Mediator of Ultra-Rapid Mortality in Sepsis and Acute Lung Injury**

**Authors:** Jianyu Wang^1,2,†^, Zhongxing Xu^1,†^, Lin Wang^1^, Xin Sui^1^, Yuan Luo^1*^, Xiuli Zhao^2*^, Jun Yang^1*^, Yongan Wang^1*^

**Affiliations:**

^1^Academy of Military Medical Sciences, Beijing, 100850, China

^2^Shenyang Pharmaceutical University, Shenyang, 117004, China

*Corresponding author. Email: ajaway@126.com, yonganw@126.com, luoyuan2006@163.com, raura3687yd@163.com

† These authors contributed equally to this work.

**Method**

**1 Material**

PLA2G2A (Human, UniProt ID: P14555), PLA2G2D (Human, UniProt ID: Q9UNK4), Varespladib (CAS: 172732-68-2), and Aristolochic acid C (CAS: 4849-90-5) were purchased from MedChemExpress (MCE, USA). PLA2G3 (Human, UniProt ID: Q9NZ20) and PLA2G5 (Human, UniProt ID: P39877) were obtained from Beijing Anxinkang Biotechnology Co., Ltd. PLA2G3 (Bee-derived, UniProt ID: P00630), 1-Oleoyl-2-hydroxy-sn-glycero-3-phospho-L-serine (18:1 Ly-PS, CAS: 326589-90-6), 1-Palmitoyl-2-hydroxy-sn-glycero-3-phospho-L-serine (16:0 Ly-PS, CAS: 143077-66-1), 1-Palmitoyl-2-hydroxy-sn-glycero-3-phosphoethanolamine (16:0 Ly-PE, CAS: 53862-35-4), 1-Palmitoyl-2-hydroxy-sn-glycero-3-phosphocholine (16:0 Ly-PC, CAS: 17364-16-8), Palmitic acid (P, CAS: 57-10-3), and Oleic acid (O, CAS: 112-80-1) were purchased from Merck (Germany). Stearic acid (S, CAS: 57-11-4) and Myristoleic acid (M, CAS: 544-64-9) were supplied by Shanghai Aladdin Biochemical Technology Co., Ltd. 1,2-Dimyristoyl-sn-glycero-3-phospho-L-serine (DMPS, CAS: 321595-13-5), 1,2-Distearoyl-sn-glycero-3-phospho-L-serine (DSPS), 1,2-Dimyristoyl-sn-glycero-3-phosphoethanolamine (DMPE, CAS: 998-07-2), 1,2-Distearoyl-sn-glycero-3-phosphoethanolamine (DSPE, CAS: 1069-79-0), 1-Myristoyl-2-palmitoyl-sn-glycero-3-phosphocholine (MPPC, CAS: 69525-80-0), 1,2-Dimyristoyl-sn-glycero-3-phosphocholine (DMPC, CAS: 18194-24-6), 1,2-Dioleoyl-sn-glycero-3-phospho-L-serine (DOPS, CAS: 90693-88-2), 1,2-Dipalmitoyl-sn-glycero-3-phospho-L-serine (DPPS, CAS: 145849-32-7), 1-Stearoyl-2-oleoyl-sn-glycero-3-phosphocholine (SOPC, CAS: 56421-10-4), and 1,2-Dihexadecanoyl-sn-glycero-3-phosphocholine (DPPC, CAS: 63-89-8) were purchased from Xi’an Ruixi Biological Technology Co., Ltd. 1,2-Dioleoyl-sn-glycero-3-phosphoethanolamine (DOPE, CAS: 4004-05-1) and 1,2-Dipalmitoyl-sn-glycero-3-phosphoethanolamine (DPPE, CAS: 923-61-5) were obtained from Corden Pharma.

**2 Evaluation of pulmonary barrier function**

I. Molecule Permeability Assessment Under Pulmonary Injury Conditions:

C57BL/6N mice were divided into two injury models: (1) acute lung overinflation induced by PLA2 (50U) via IT administration, (2) inflammatory injury induced by LPS (1mg/mL) via IT administration. Barrier permeability was assessed using Evans blue dye (2%) administered via two routes respectively: (a) IT administration (50 µL) and (b) IV tail vein injection (200µL). At 0.5 h post-dye delivery, lungs were collected for fluorescence histopathological analysis to evaluate Evans blue extravasation area.

II. PLA2 penetration in Disease-Specific pulmonary barrier

FITC-labeled PLA2 was prepared by reacting 1 mg FITC with 50 U PLA₂ in PBS (pH 7.4) under light-protected conditions at room temperature for 4 h, followed by purification using 10 kDa centrifugal filters and PBS washes 3 times to remove unreacted FITC. Cy7-labeled PLA2 was prepared using the same method with NHS-Cy7 ester.

For in vivo assessment, ALI and sepsis mice were IV injected with FITC-PLA2 or Cy7-PLA2 (0.05 mg/mL), respectively, followed by in vivo fluorescence imaging (IVIS Spectrum) at specified timepoints (ALI: 4/24/48 h; sepsis: 5/10 h, via Cy7-labeled PLA2) and fluorescence histopathological evaluation (via FITC-labeled PLA2).

**3 Histological analysis:**

organs were collected and fixed in 4% paraformaldehyde for 48 h, dehydrated, embedded, sectioned into 4 μm slices, and stained with hematoxylin-eosin or incubated with primary/secondary antibodies (IL-6) for microscopic observation.

**4 Blood pressure measurement in mice**

Mice were placed in an intelligent non-invasive blood pressure system (Beijing Zhongshi Dichuang Technology Co., Ltd.) within 30 min post-administration. Systolic blood pressure (SBP), diastolic blood pressure (DBP), and heart rate (HR) were measured via tail-cuff method.

**5 Blood gas analysis**

Anesthetized mice were administered drugs via atomizing needle or ultrasonic nebulizer (Jinan Yiyan Technology Development Co., Ltd.). After 1 and 24 h, aortic blood was collected and analyzed using a blood gas analyzer (Shanghai Radiometer Medical Equipment Co., Ltd.) to monitor PaO_2_ and PaCO_2_ changes.


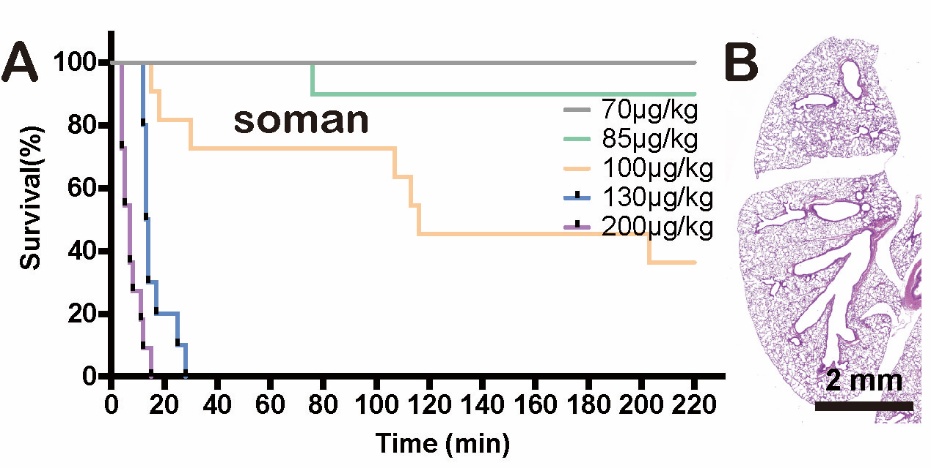


**Figure S1**. A) Survival time of mice treated with soman (*n*=10). B) H&E histopathology of mice treated with soman.


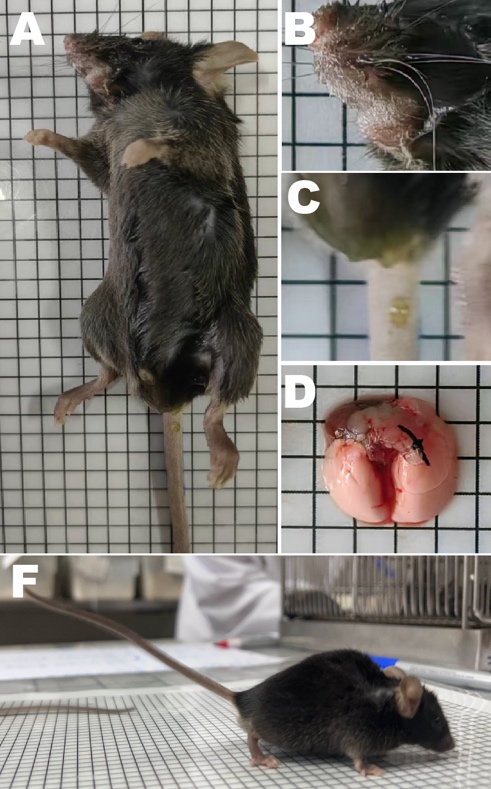


**Figure S2.** Photographs of soman intoxication in animals. A) Whole-body presentation of intoxicated mouse. B) Profuse salivation. C) Urinary incontinence. D) Lung showing absence of hemorrhagic lesions. E) Tail rigidity and spasms.


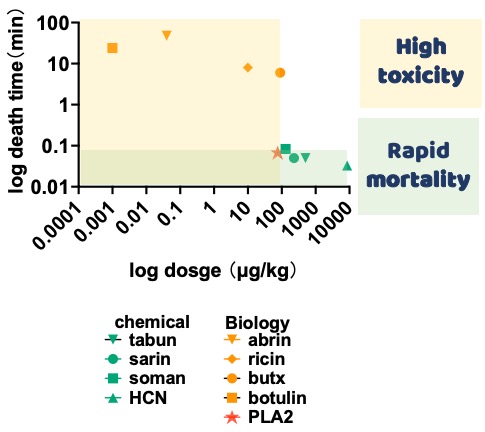


**Figure S3**. The death time and lethal dose (LD_50_) of classical chemical and biology toxicant.


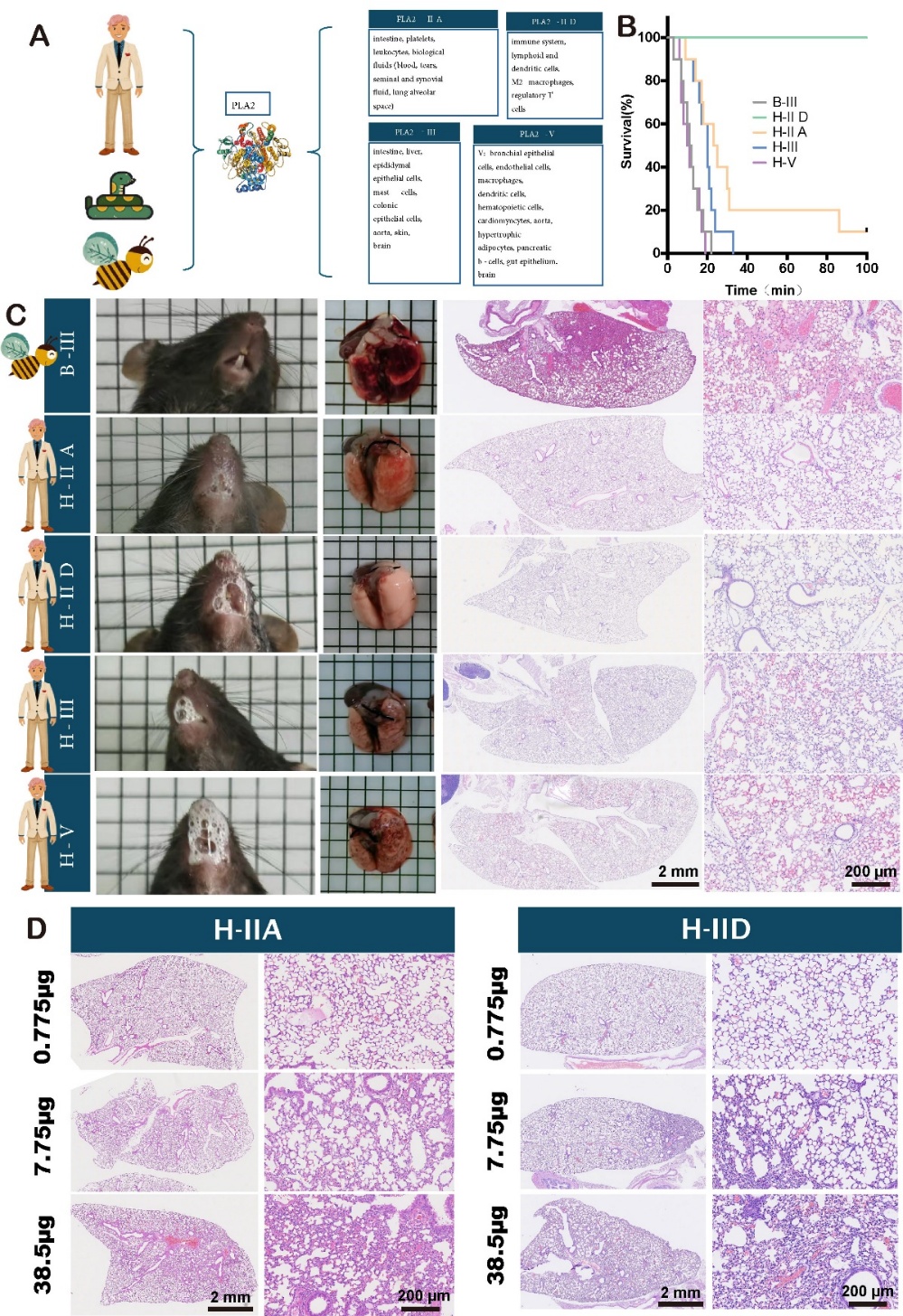


**Figure S4**. A) Schematic of PLA2 diversity by source, subtype, and secretory origin. B–C) Survival curves (B, *n*=10) and lung photographs (C; left) with H&E histopathology (C; right) in mice treated with distinct PLA2 subtypes. D) Dose-dependent pulmonary histopathology induced by human PLA2-IIA and PLA2-IID. H-IIA, Humanized PLA2-IIA; H-IID, Humanized PLA2-IID; H-III, Humanized PLA2-III; H-V, Humanized PLA2-V; B-III, bee venom-derived PLA2-III.


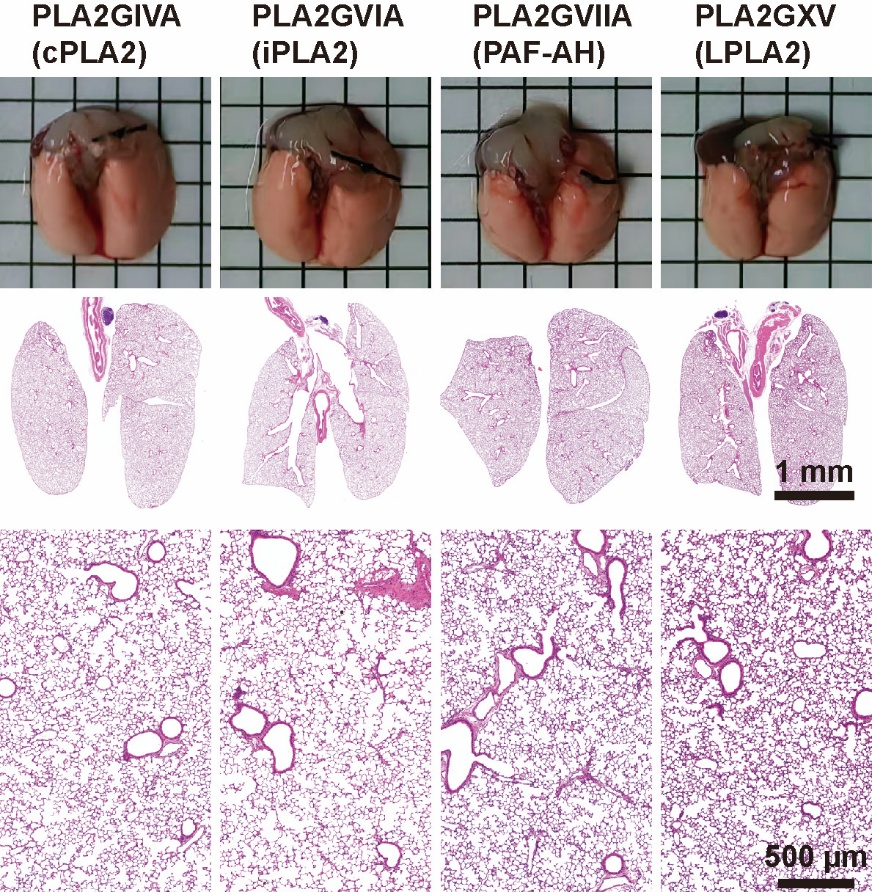


**Figure S5.** Lung photographs and H&E-stained histopathological images of mice treated with human PLA2GIVA, PLA2GVIA, PLA2GVIIA, and PLA2GXV.


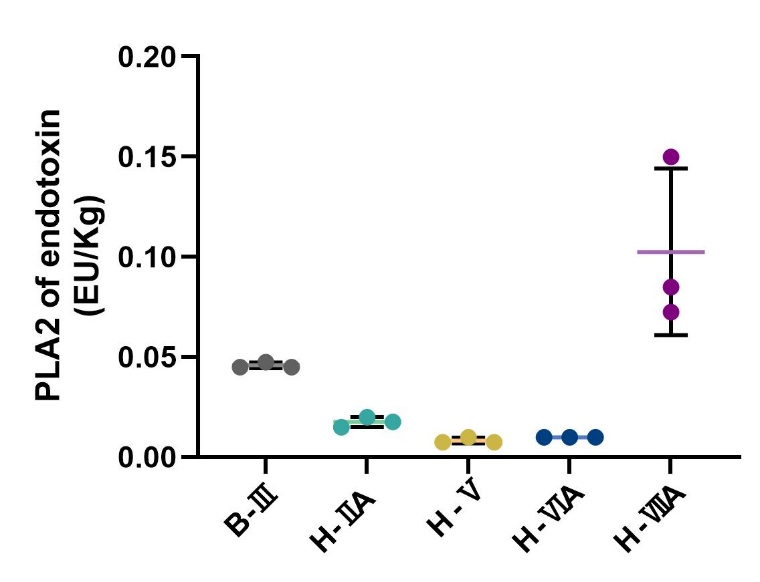


**Figure S6.** Endotoxin content of Bee PLA2GIII, and human PLA2GIIA, PLA2GV, PLA2GVIA, and PLA2GVIIA (*n*=3).


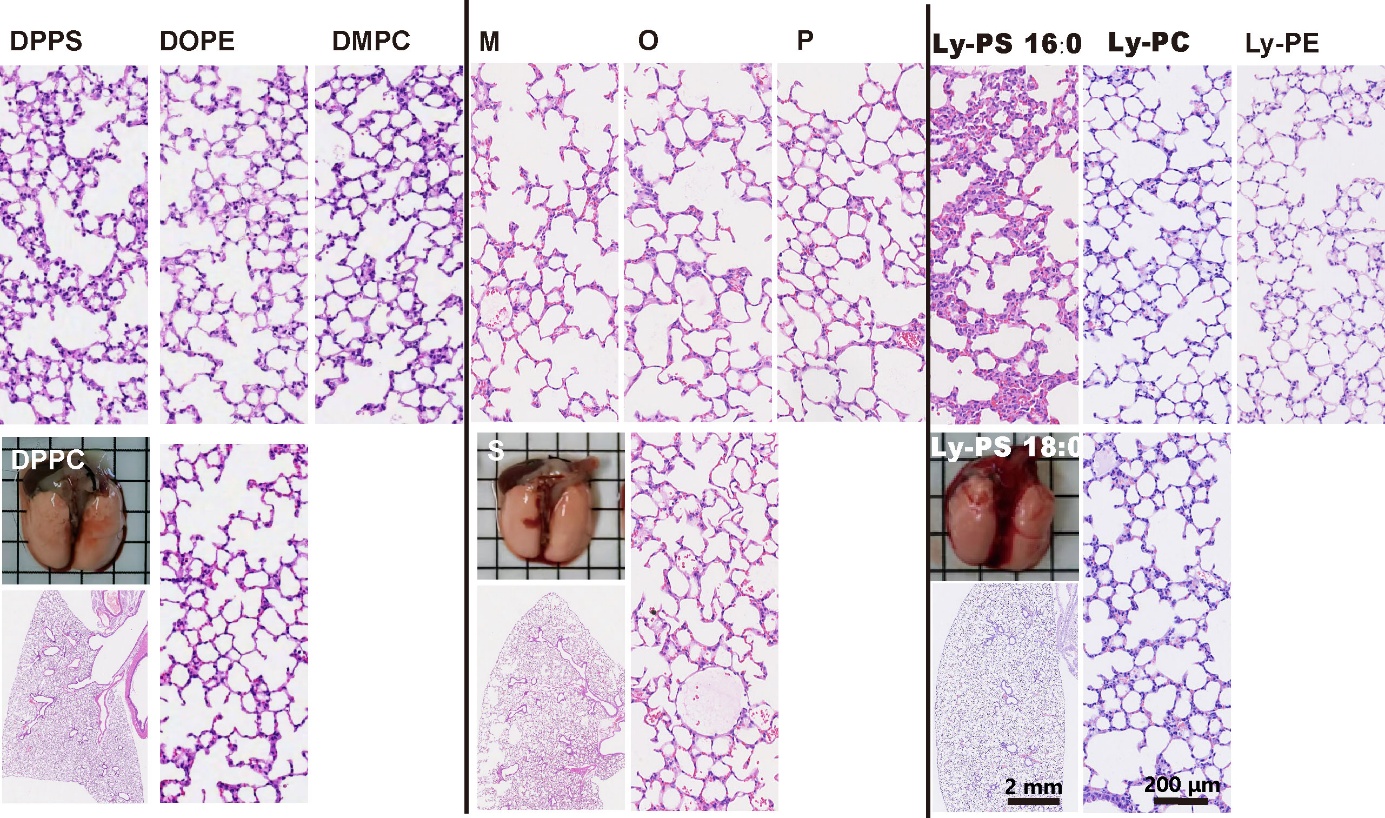


**Figure S7**. photographs and histopathology of lungs administrated (IT) with PLs (left), FA (Myristic acid, M; Oleic acid, O; Palmitic acid, P) (middle) and lyso-PLs (right)(*n*=3).


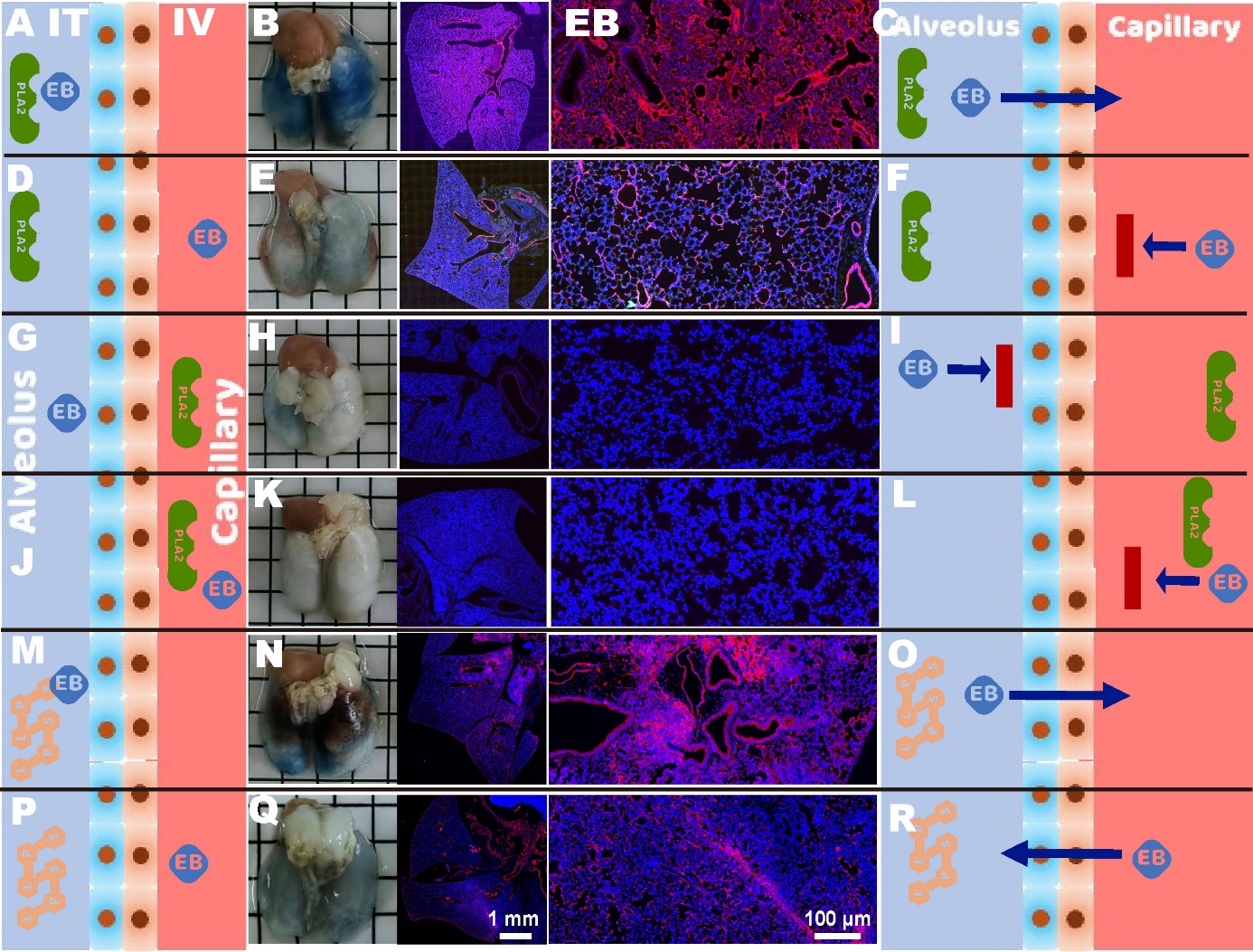


**Figure S8.** A-F) EB penetration under PLA2 IT administration: administration schematics (A-IT, D-IV), EB diffusion photographs/fluorescence histopathology (B-IT, E-IV), and penetration schematics (C-IT, F-IV). G-L) EB penetration under PLA2 IV administration: administration schematics (G-IT, J-IV), EB diffusion photographs/fluorescence histopathology (H-IT, K-IV), and penetration schematics (I-IT, L-IV). M-R) EB penetration under LPS IT administration: administration schematics (M-IT, P-IV), EB diffusion photographs/fluorescence histopathology (N-IT, Q-IV), and penetration schematics (O-IT, R-IV). ALI, acute lung injury; EB, Evans blue; LPS, lipopolysaccharide.


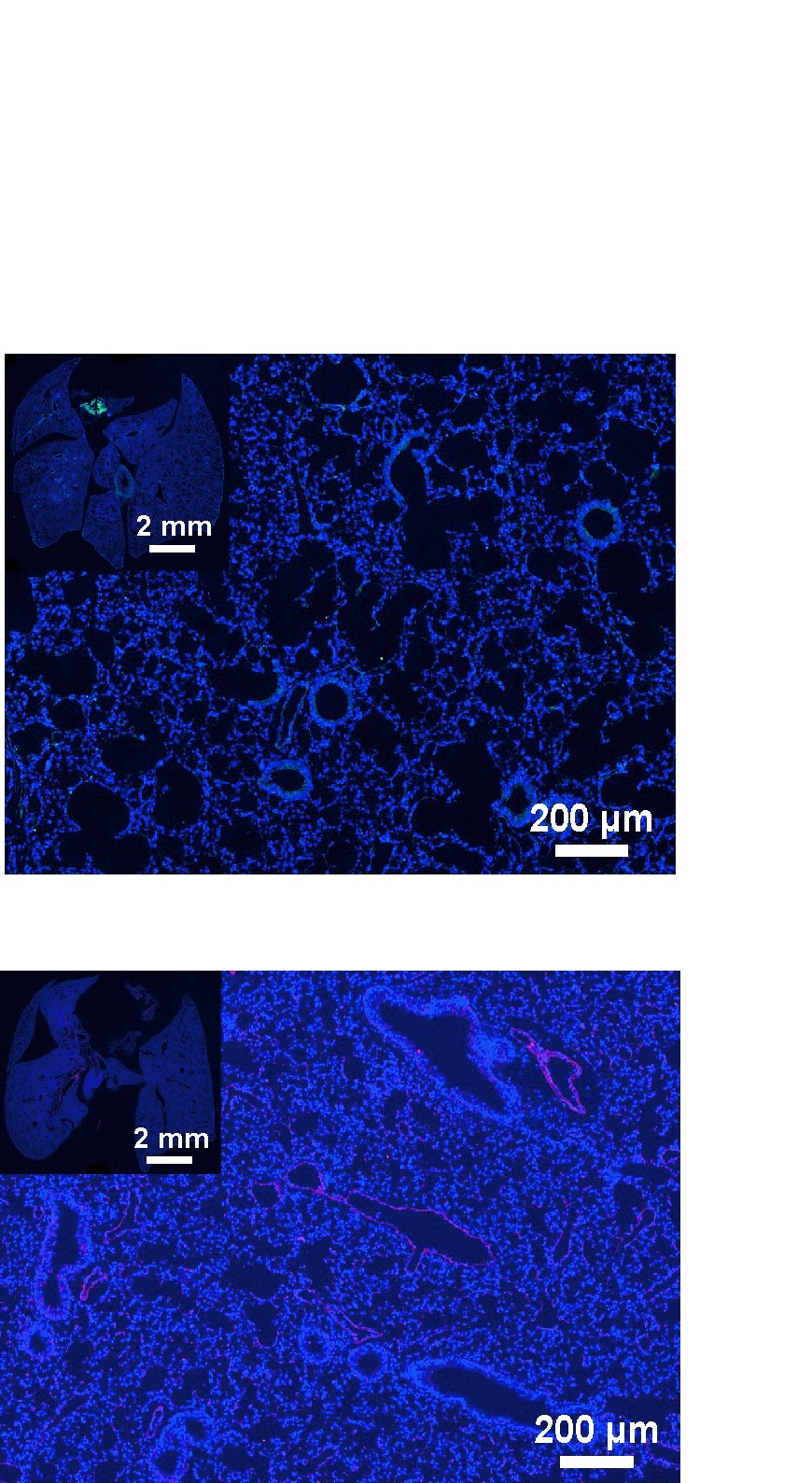


**Figure S9**. Fluorescence images of lung section from mice treated with EB IV injection after soman exposure. (Blue: DAPI stain, Red: EB)


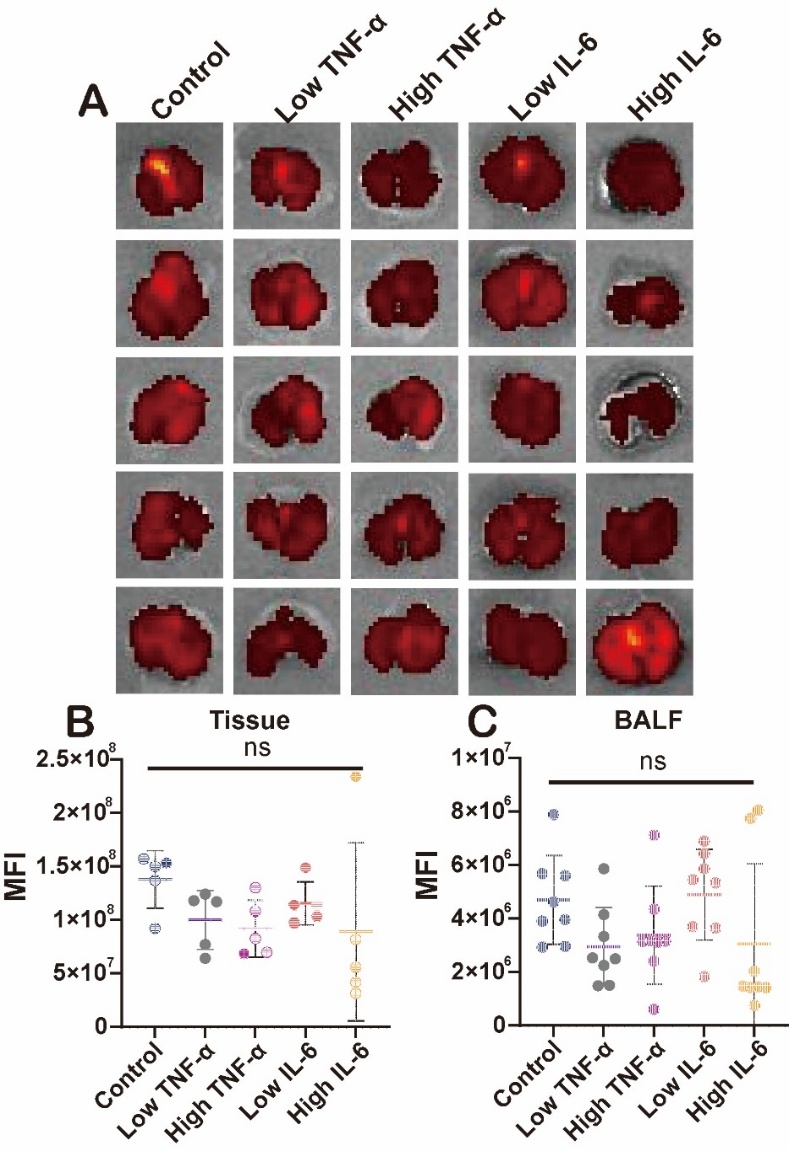


**Figure S10.** Effect of TNF-α and IL-6 via intratracheal (IT) administration on pulmonary barrier, in vivo tracking of FITC labeled PLA2 (A, *n*=5), and MFI in lung tissue (B, *n*=5) and BALF (C, *n*=8). High: 0.42 ng/mouse; Low: 0.042 ng/mouse.


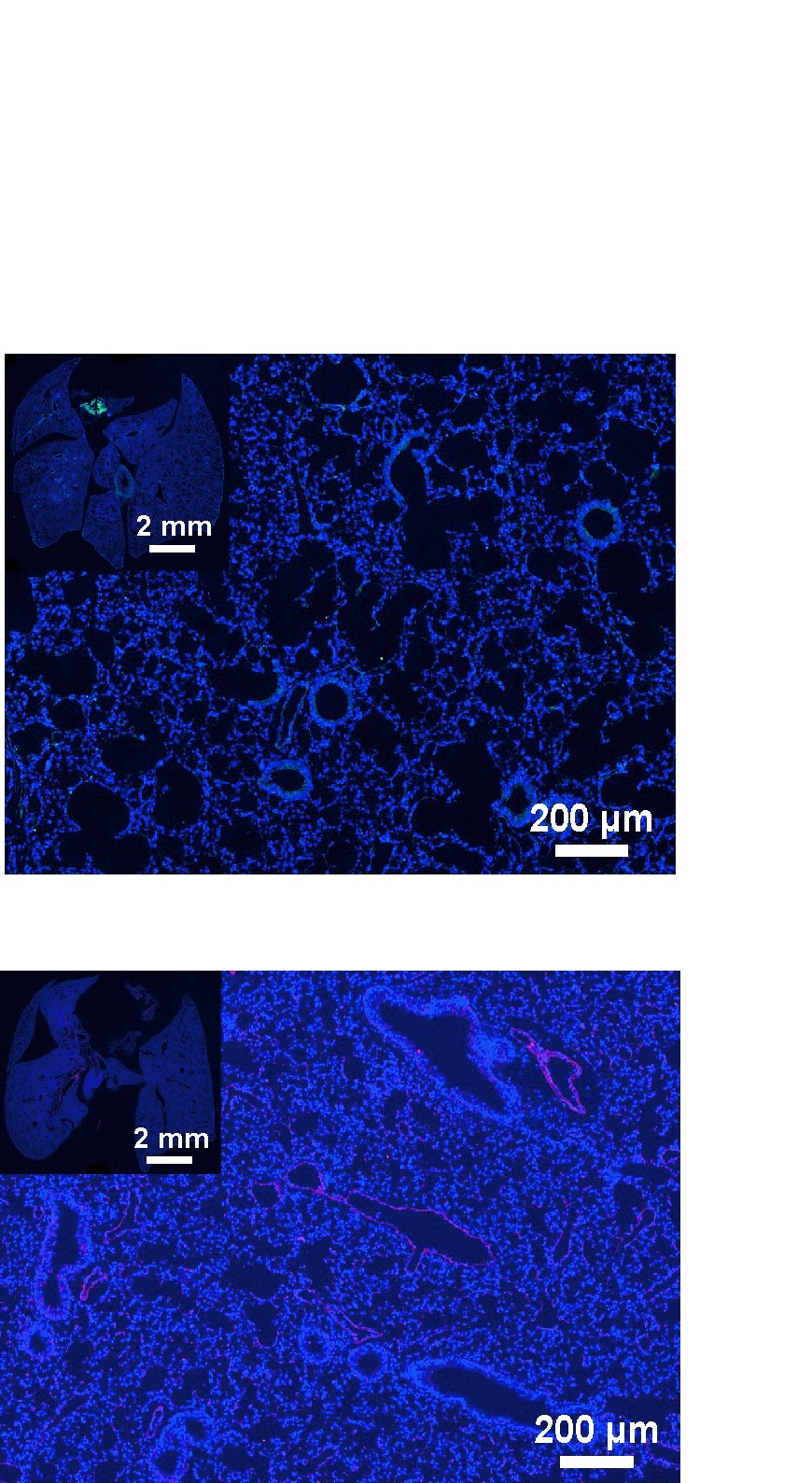


**Figure S11**. Fluorescence images of lung section from mice treated with FITC-PLA2 IV injection after soman exposure. (Blue: DAPI stain, Green: FITC)


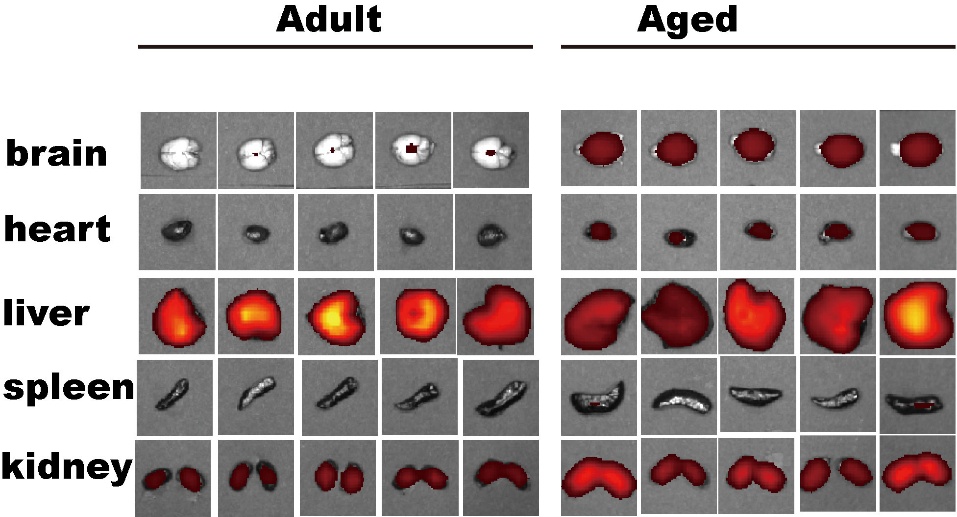


**Figure S12**. Fluorescence in vivo images of Cy7 labeled PLA2 in main organs form adult and aged mice(*n*=5).


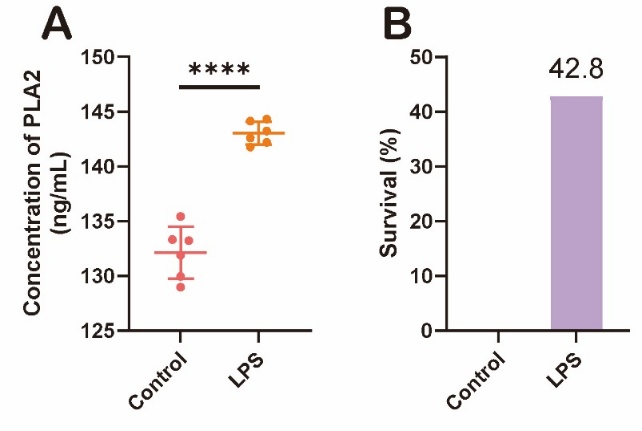


**Figure S13.** Concentration of PLA2 in blood (A, *n*=6, *****p* < 0.0001) and survival rate (B) in aged mice with ALI.


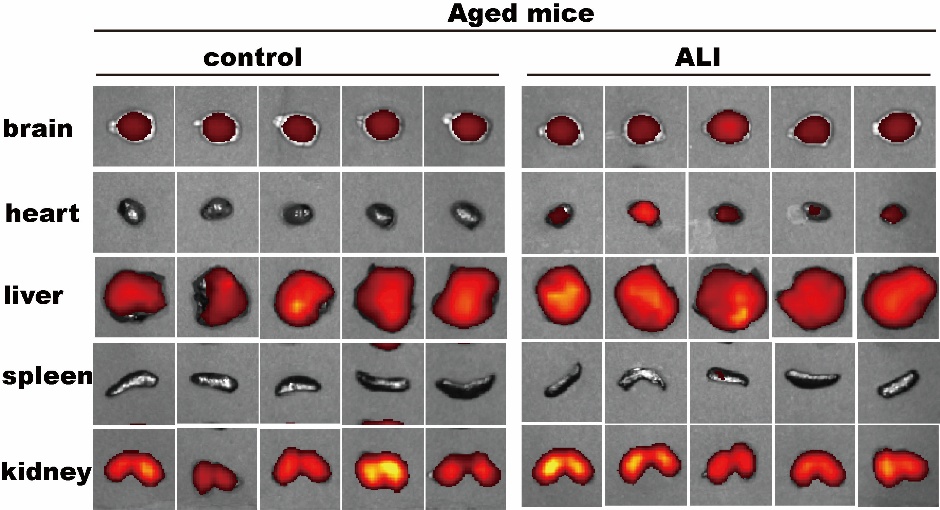


**Figure S14**. Fluorescence in vivo images of Cy7 labeled PLA2 in main organs form aged mice with ALI (*n*=5).


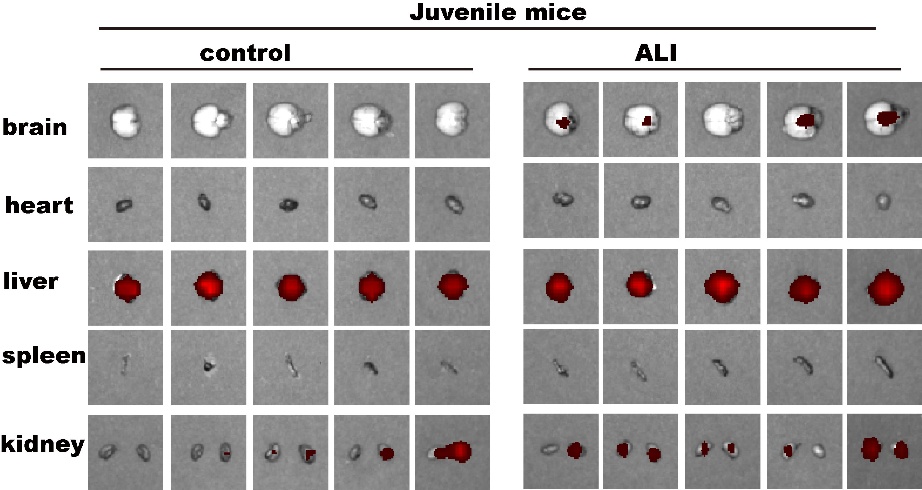


**Figure S15**. Fluorescence in vivo images of Cy7 labeled PLA2 in main organs form juvenile mice with ALI (*n*=5).


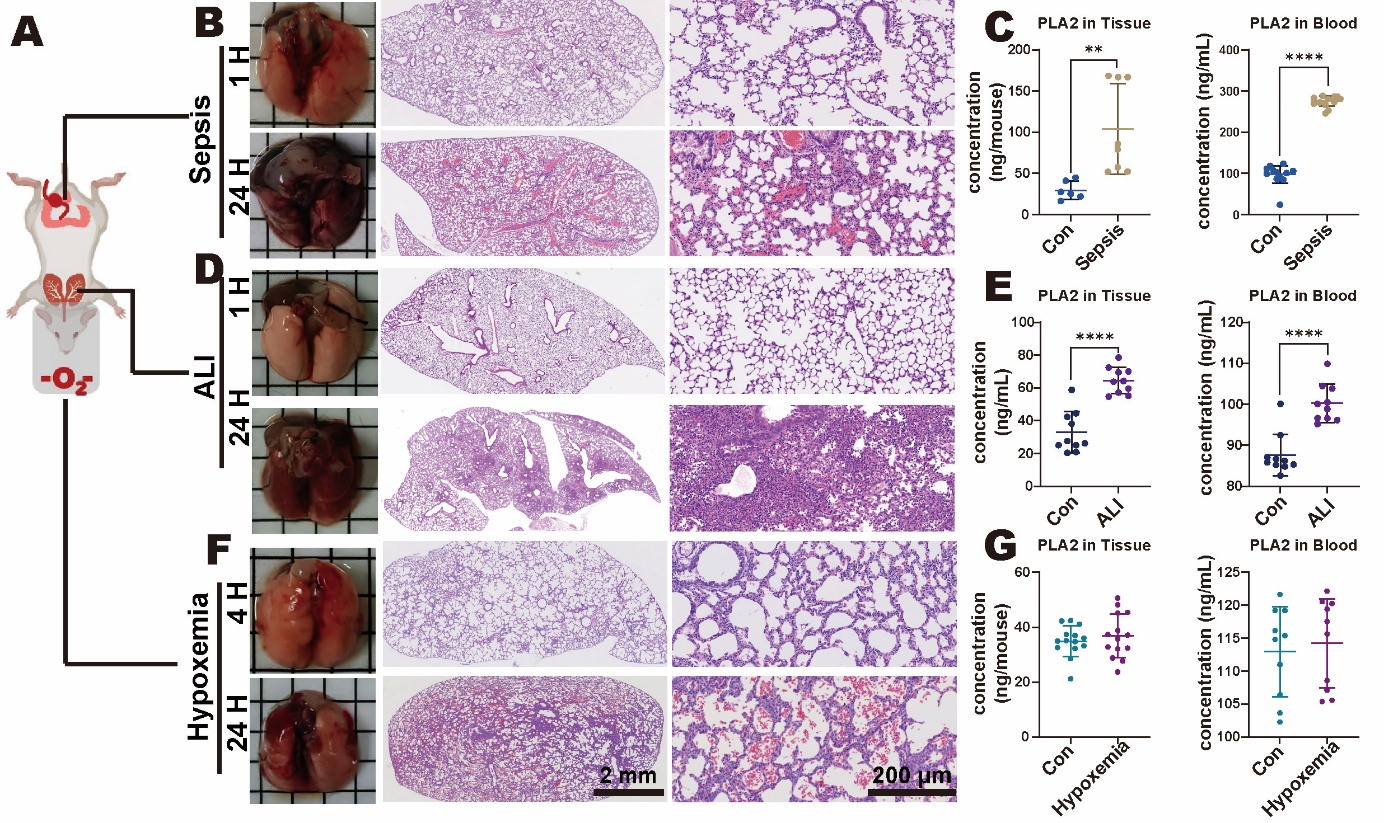


**Figure S16.** A) Schematic of sepsis, ALI and hypoxia mouse models. B, D, F) Lung injury progression in sepsis (B), ALI (D) and hypoxemia (F) models: photographs (left) and histopathology (right). C, E, G) PLA2 levels in lung tissue (left) and blood (right) at 24h in sepsis (C, tissue: con, *n*=6; sepsis, *n*=8. Blood: *n*=12; ****p* < 0.001, *****p* < 0.0001), ALI (E, tissue: *n*=10, blood: *n*=10, *****p* < 0.0001) and hypoxemia (G, tissue: *n*=14, blood: *n*=10) models.

**Figure S17**. In vivo stability of PLA2 administered intravenously at 2 ng per mouse (*n*=8).

**Figure S18**. Survival rates of ALI, septic, and hypoxic mice over 24 h.


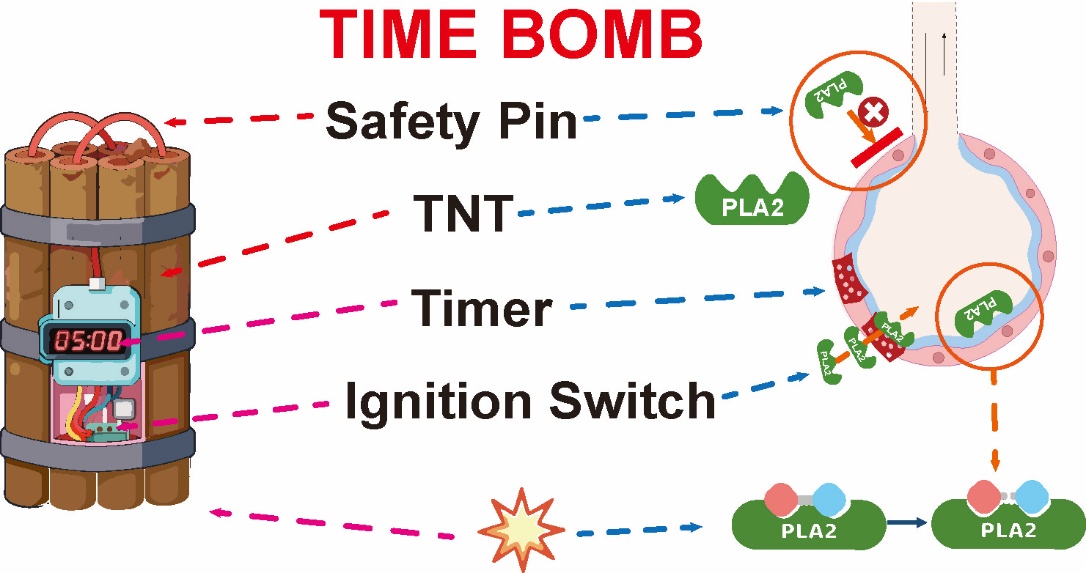


**Figure S19**. Schematic of the pulmonary barrier safety mechanism.


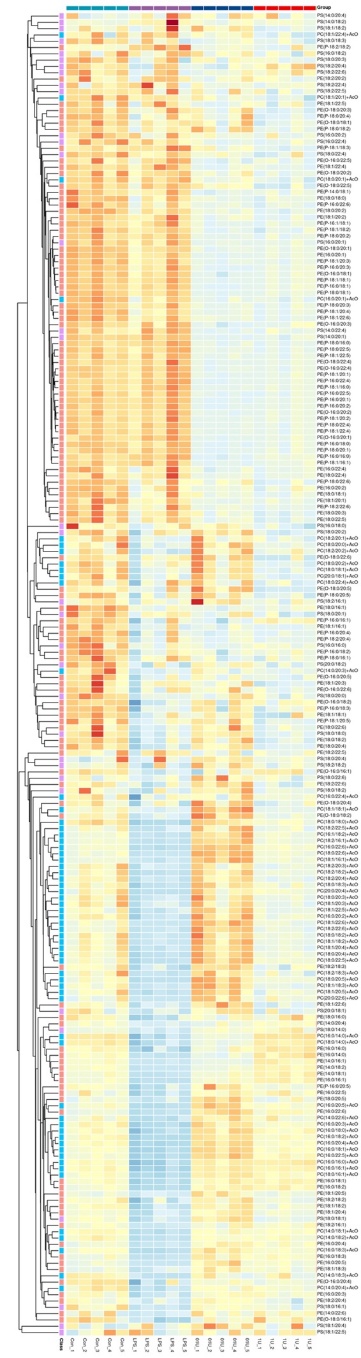

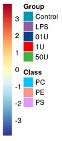


**Figure S20**. Heatmap of PLs in BALF from mice exposed to ALI and PLA2(*n*=4).


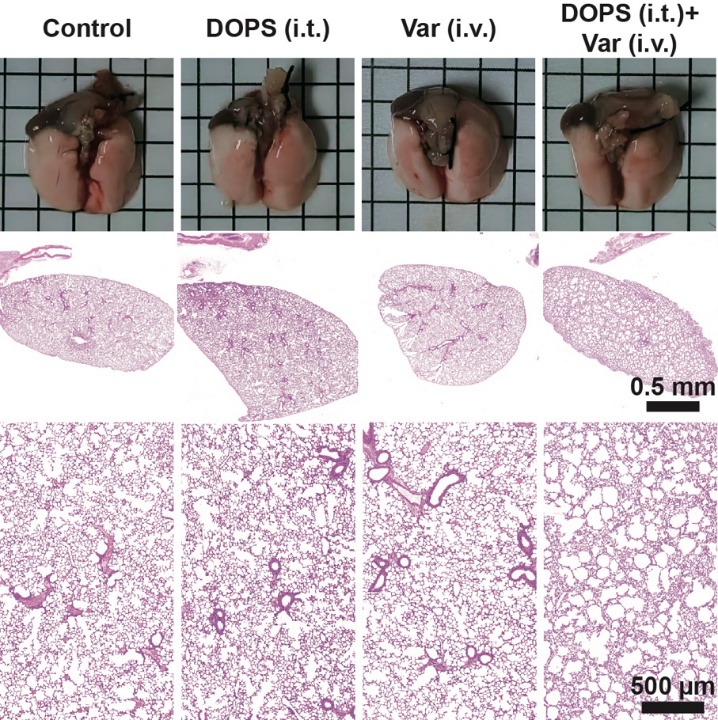


**Figure S21**. H&E histopathology of lungs form mice treated with Var and DOPS. Insert: lung photograph.

**Table S1** Mortality or significant lung injury induced by different PLA2s following intratracheal instillation.

|  |  |  | Rapid Death |  | Moderate Damage |  | Safe |
| --- | --- | --- | --- | --- | --- | --- | --- |
| sPLA2 | GIII |  | Yes |  |  |  |  |
|  | GV |  | Yes |  |  |  |  |
|  | GIIA |  |  |  | Yes |  |  |
|  | GIID |  |  |  |  |  | Yes |
| cPLA2 | GIVA |  |  |  |  |  | Yes |
| iPLA2 | GVIA |  |  |  |  |  | Yes |
| PAF-AH | GVIIA |  |  |  |  |  | Yes |
| LPLA2 | GXV |  |  |  |  |  | Yes |

**Table S2** Decrease in various phospholipids in the lung after intratracheal administration of PLA2.

|  |  |  | control（%） | | |  | PLA2（%） | | | | | | Decrease（%） | | | | |
| --- | --- | --- | --- | --- | --- | --- | --- | --- | --- | --- | --- | --- | --- | --- | --- | --- | --- |
| PE | P-16:0/18:1 PE |  | 1.203 | ± | 0.176 |  | | 0.417 | ± | 0.054 | |  | | 65.34 | | *** | |
|  | P-16:0/20:4 PE |  | 0.703 | ± | 0.04 |  | | 0.476 | ± | 0.067 | |  | | 32.29 | | ** | |
|  | P-16:0/22:4 PE |  | 0.516 | ± | 0.061 |  | | 0.131 | ± | 0.042 | |  | | 74.61 | | *** | |
|  | PLPE |  | 0.486 | ± | 0.08 |  | | 0.215 | ± | 0.017 | |  | | 55.76 | | ** | |
|  | POPE |  | 0.33 | ± | 0.05 |  | | 0.417 | ± | 0.054 | |  | | none | |  | |
|  | P-16:0/18:2 PE |  | 0.297 | ± | 0.028 |  | | 0.215 | ± | 0.017 | |  | | 27.61 | | *** | |
| PC | DPPC |  | 2.15 | ± | 0.237 |  | | 2.617 | ± | 0.285 | |  | | none | |  | |
|  | PPPC |  | 1.519 | ± | 0.2 |  | | 1.826 | ± | 0.265 | |  | | none | |  | |
|  | PLPC |  | 0.798 | ± | 0.106 |  | | 0.858 | ± | 0.127 | |  | | none | |  | |
|  | POPC |  | 0.726 | ± | 0.094 |  | | 0.858 | ± | 0.108 | |  | | none | |  | |
|  | PAPC |  | 0.351 | ± | 0.031 |  | | 0.365 | ± | 0.055 | |  | | none | |  | |
|  | PMPC |  | 0.349 | ± | 0.036 |  | | 0.406 | ± | 0.042 | |  | | none | |  | |
| PS | DSPS |  | 0.0727 | ± | 0.021 |  | | 0.039 | ± | 0.004 | |  | | 46.35 | | ** | |
|  | SOPS |  | 0.0235 | ± | 0.001 |  | | 0.019 | ± | 0.003 | |  | | 19.15 | | * | |
|  | LAPS |  | 0.007 | ± | 0.002 |  | | 0.008 | ± | 0.002 | |  | | none | |  | |
|  | LPPS |  | 0.006 | ± | 0.002 |  | | 0.004 | ± | 0.001 | |  | | 33.33 | |  | |
|  | SPPS |  | 0.005 | ± | 0.001 |  | | 0.003 | ± | 0.001 | |  | | 40.00 | |  | |
|  | PSPS |  | 0.004 | ± | 0.002 |  | | 0.002 | ± | 0.001 | |  | | 50.00 | |  | |
|  |  |  |  |  | * *p* < 0.05, ** *p* < 0.01, *** *p* < 0.001 | | | | | |  | | | |  | |  |

**Table S3** Binding rate of PLA2 to various phospholipids.

|  | POPS | DOPS | DMPS | DPPS | DSPS | DMPC | SOPC |
| --- | --- | --- | --- | --- | --- | --- | --- |
| Binding Rate （1/M.S） | 112 | 80.7 | 80.2 | 11.1 | 0 | 0 | 0 |
|  | MPPC | DSPC | DPPC | DOPE | DPPE | DSPE | DMPE |
| Binding Rate （1/M.S） | 0 | 0 | 0 | 0 | 0 | 0 | 0 |
